# Operational and Environmental Stability Assessment of Silicon and Copper Phthalocyanine‐Based OTFTs

**DOI:** 10.1002/smtd.202500782

**Published:** 2025-08-15

**Authors:** Nicholas J. Dallaire, Joonhyung Park, Raluchukwu B. Ewenike, Halynne R. Lamontagne, Chang‐Hyun Kim, Benoît H. Lessard

**Affiliations:** ^1^ School of Electrical Engineering and Computer Science University of Ottawa 800 King Edward Ave Ottawa ON K1N6N5 Canada; ^2^ School of Electronic Engineering Gachon University Seongnam 13120 Republic of Korea; ^3^ Department of Chemical and Biological Engineering University of Ottawa 161 Louis Pasteur Ottawa ON K1N6N5 Canada

**Keywords:** copper phthalocyanine, device modeling, OTFT, Raman microscopy, silicon phthalocyanine, stability

## Abstract

When developing new materials for organic electronics, understanding how they will perform and change over time is critical. Typical bias stress exposure experiments provide limited information on the materials’ performance in applications which involve multiple charging and discharging steps. Here, organic thin film transistors (OTFTs) are characterized for 48–72 h straight in air and in N_2_ using newly developed cyclic testing protocols that enable statistically significant evaluation of four different semiconductors by quantifying both, environmental and operational stress on their performance. It is demonstrated that the structure of the phthalocyanine leads to significant differences in response to bias stress, such as silicon bis(pentafluorophenoxy)phthalocyanine (F_10_‐SiPc) showing a much more air‐stable *p*‐type device compared to copper phthalocyanine (CuPc) and bis(pentafluorophenoxy) hexadecafluoro silicon(iv) phthalocyanine  (F_5_PhO)_2_‐F_16_‐SiPc showing much more air‐stable *n*‐type performance compared to Copper(II) 1,2,3,4,8,9,10,11,15,16,17,18,22,23,24,25‐hexadecafluoro‐29H,31H‐phthalocyanine (F_16_‐CuPc). Raman microscopy of the films revealed no changes in morphology. The devices are also modeled using the 2D finite‐element method, which suggests that most changes in device performance are due to fixed charges at the semiconductor/insulator interface. Overall, OTFT stress testing demonstrates, that important structure property relationships can be established between semiconductor molecular structure and device performance.

## Graphical abstract

Time saving Automation: Environmental and Cycling stress tests in phthalocyanines based organic thin film transistors.

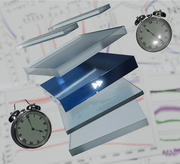

## 1 Introduction

Organic thin film transistors (OTFTs) have gained notable traction over the years, in part due to their vast number of uses as biosensors,^[^ ^1^ ^]^ gas sensors,^[^ ^2^ ^]^ photo detectors,^[^ ^3^, ^4^ ^]^ and flexible and printable circuit elements,^[^ ^3^, ^5^ ^]^ which is well paired with their potential to be biocompatible.^[^ ^6^, ^7^, ^8^ ^]^ OTFTs are often characterized using metal‐oxide‐semiconductor field‐effect transistor (MOSFET) equations for the evaluation of threshold voltage (*V_T_*), carrier mobility (*µ*), *I_ON_/I_OFF_* ratio and more, while some new models are being developed to better account for contact resistance and voltage dependent effects,^[^ ^9^, ^10^ ^]^ these models are less commonly employed in the literature. Using novel morphologies,^[^ ^11^ ^]^ semiconductor‐dielectric combinations,^[^ ^12^, ^13^, ^14^ ^]^ electrode matching,^[^ ^15^, ^16^ ^]^ and other methods, these parameters can be optimized for a particular application. The key parameters are often evaluated immediately after OTFT fabrication, which gives a good estimate of the peak performance but lacks a full understanding of their stability. To make OTFTs viable in real‐world applications, it is crucial to understand their short‐term and long‐term stability in various environments. Poor OTFT stability is often manifested through a shift in the *V_T_*, an increase or decrease in *µ*, and typically an increase in the off current, resulting in a worse *I_ON_/I_OFF_* ratio.^[^ ^17^ ^]^ The main factors behind the instability of OTFTs are changes to the dipolar orientation of the molecules and the percolation of moisture, oxygen, or other impurities through pores in the semiconductor or dielectric layer, causing charge trapping and de‐trapping events.^[^ ^18^, ^19^ ^]^ The majority of studies, that focus on stability, are executed by an initial device test, followed by leaving the device in air for a determined amount of time before testing again, or testing devices at set voltages and measuring changes in relative current. For instance, Boileau et al. studied the stability of electrical performance on metal phthalocyanines based OTFTs while exposed to various temperatures and environments by applying a constant voltage or cycling at various temperatures.^[^ ^20^ ^]^ Yang et al. evaluated the change in performance by evaluating current decay curves over time.^[^ ^21^ ^]^ Many other papers, including those from our group, show results from similar stability tests.^[^ ^18^, ^22^, ^23^, ^24^, ^25^ ^]^ These studies are valuable in terms of assessing the effect of the environment, but lack information on the operational stability when the device is continuously cycled. This information is crucial in applications where devices are pulsed or cycled rather than continuously biased, such as in LED display controllers, motor speed controllers, speaker amplifiers, and heating element controllers.^[^ ^26^ ^]^

**Figure 1:**
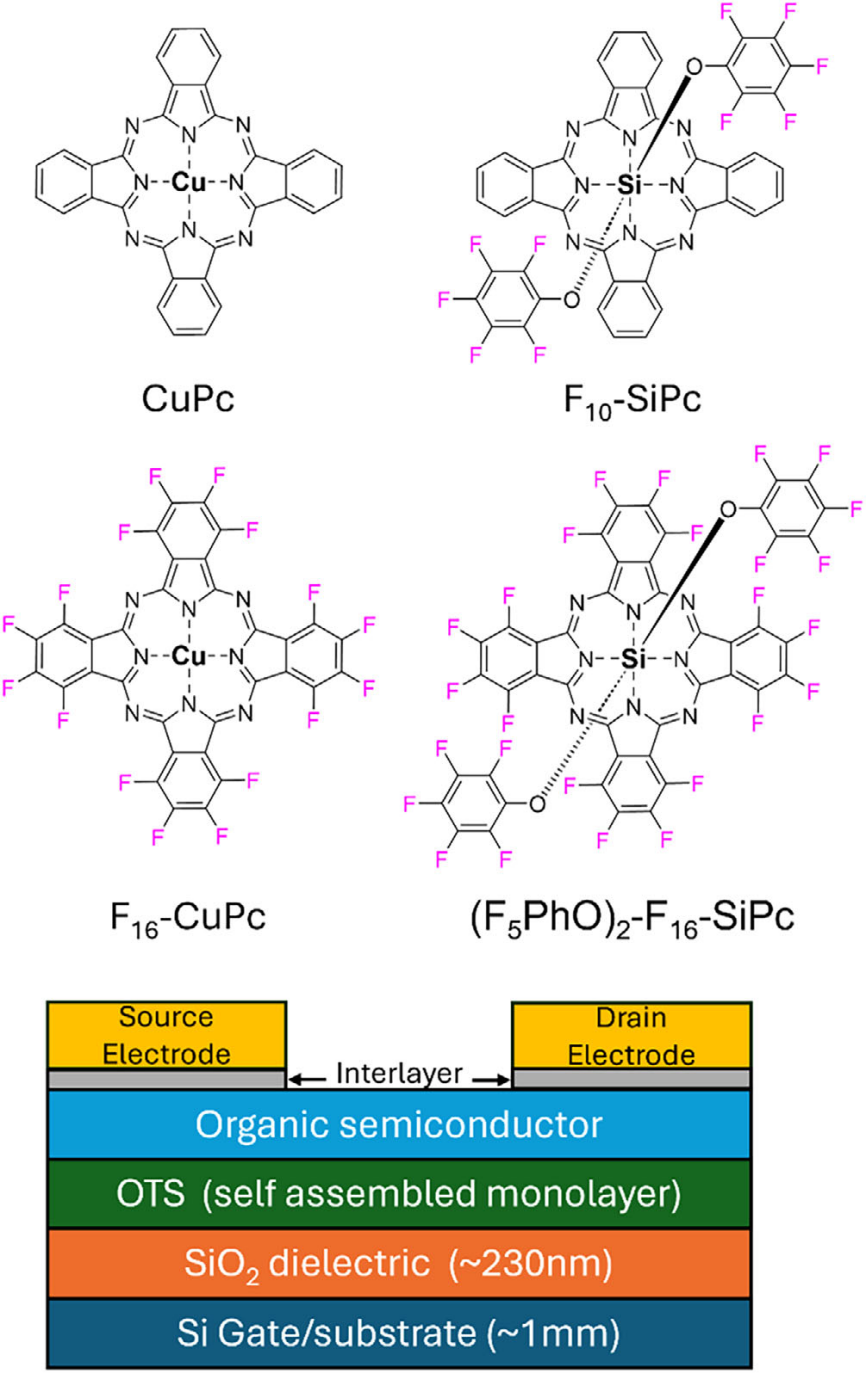
Chemical structures of the organic semiconductors used in this study (CuPc, F_10_SiPc, F_16_CuPc, and (F_5_PhO)_2_‐F_16_‐SiPc), along with the OTFT device structure.

In this study, we evaluated the operational and environmental stability of common and more recently developed copper and silicon phthalocyanines based OTFTs (**Figure** **1**): Copper phthalocyanine (CuPc), Copper(II) 1,2,3,4,8,9,10,11,15,16,17,18,22,23,24,25‐hexadecafluoro‐29H,31H‐phthalocyanine (F_16_CuPc), silicon bis(pentafluorophenoxy)phthalocyanine ((F_5_PhO)_2_‐SiPc or F_10_SiPc) and bis(pentafluorophenoxy) hexadecafluoro silicon(iv) phthalocyanine ((F_5_PhO)_2_‐F_16_‐SiPc) based OTFTs in a bottom gate top contact geometry. We use several of our in‐house designed autotesters to continuously test over 20 devices every 40 min over multiple days with environmental control.^[^ ^27^ ^]^ The devices containing either a *p*‐channel organic semiconductors (referred to as *p*‐type) or an *n*‐channel organic semiconductor (referred to as *n*‐type) were tested in air and in N_2_ over 2500 min. We decoupled the effect of operational stability from environmental stability and modeled the performance to highlight the effect of donor/acceptor‐like exponential trap density, temperature, and fixed interface charge density (*N_int_*). Most devices experienced a shift in performance after only a few hours before finally saturating. We also characterized the films before and after the operation. Our findings suggest that no change in morphology or molecular orientation was observed and that changes in performance are dominated by the changes in *N*_*int*_, even in a controlled *N_2_* environment. We demonstrate that original performances are not the same as stressed performances and that these differences are important for characterizing new materials and are critical for the deployment of OTFTs in real applications.

## 2 Results and Discussion

Bottom gate top contact OTFTs were fabricated on OTS‐treated Si wafers using four different semiconductors, CuPc, F_10_SiPc, F_16_CuPc, and (F_5_PhO)_2_‐F_16_‐SiPc (Figure 1), and were characterized under different conditions and environments. Each semiconductor was characterized right after being fabricated, both in air and/or *N_2_* (*Baseline*, **Table** **1**). The same devices were then either continuously cycled as an OTFT in the same conditions for 48–72 h (*Cycled*, Table 1) or simply sat in the same environment as those cycled and were tested after the same 48–72 h, then re‐characterized (*Env. Control*, Table 1).

**Table 1:** Summary of the OTFT electrical characteristics before and after stress testing.

	Env. and Type ^a)^	Test type ^a)^	*V_T_* [V] ^b)^	*µ* [10^−2^ cm^2^/Vs] ^b)^	*I_ON_/I_OFF_* ^b)^	*SS* ^b)^ *[V/decade]*	*Hysteresis [V]*	*N*_int_ [cm^−2^] ^b)^
CuPc ^a)^	Air, *p*‐type	Baseline	− 0.2 ± 1.0	1.27 ± 0.08	(2.4 ± 0.3) × 10^3^	*−4.8 ± *1.0	*0.076 ± *0.046	− 9.0 × 10^11^
		Env. Control	39.8 ± 5.5	0.72 ± 0.10	(9.1 ± 0.7) × 10^0^	−50 ± 6.9	1.87 ± 2.23	− 3.5 × 10^12^
		Cycled	39.6 ± 5.7	0.86 ± 0.09	(13 ± 1.7) × 10^0^	−41 ± 9.3	0.10 ± 0.08	− 3.5 × 10^12^
	N_2_, *p*‐type	Baseline	− 15.3 ± 3.2	0.93 ± 0.13	(3.3 ± 0.6) × 10^4^	−2.6 ± 0.88	0.37 ± 0.16	5.5 × 10^11^
		Env. Control	− 18.2 ± 1.3	0.79 ± 0.09	(7.5 ± 2.2) × 10^3^	−2.8 ± 0.70	0.16 ± 0.09	8.8 × 10^11^
		Cycled	− 22.6 ± 1.7	1.05 ± 0.18	(9.4 ± 1.0) × 10^4^	−2.4 ± 0.74	0.35 ± 0.11	5.0 × 10^11^
F_10_SiPc ^a)^	Air, *p*‐type	Baseline	− 34.5 ± 0.9	0.69 ± 0.34	(7.1 ± 1.2) × 10^4^	−6.0 ± 2.1	4.9 ± 1.5	2.0 × 10^12^
		Env. Control	− 32.6 ± 2.3	2.78 ± 0.65	(1.5 ± 0.2) × 10^5^	−6.1 ± 1.3	4.7 ± 2.4	1.5 × 10^12^
		Cycled	− 31.1 ± 0.9	1.60 ± 0.14	(3.6 ± 0.3) × 10^5^	−4.0 ± 0.53	4.2 ± 0.89	1.5 × 10^12^
	N_2_, *n*‐type	Baseline	7.4 ± 1.3	11.2 ± 3.4	(1.1 ± 0.4) × 10^5^	3.4 ± 1.3	1.5 ± 0.64	− 3.1 × 10^11^
		Env. Control	8.9 ± 2.0	12.3 ± 2.8	(5.2 ± 2.1) × 10^4^	2.7 ± 0.58	1.4 ± 0.35	− 7.2 × 10^11^
		Cycled	13.5 ± 2.1	6.2 ± 2.4	(5.0 ± 1.6) × 10^5^	2.7 ± 0.38	1.3 ± 0.67	− 7.0 × 10^11^
F_16_CuPc ^a)^	Air, *n*‐type	Baseline	21.0 ± 11	0.56 ± 0.22	(2.2 ± 0.6) × 10^4^	6.9 ± 2.5	2.7 ± 2.4	− 1.0 × 10^11^
		Env. Control	6.6 ± 2.4	0.41 ± 0.07	(5.7 ± 1.7) × 10^4^	3.7 ± 1.4	3.2 ± 0.98	− 5.0 × 10^11^
		Cycled	32.9 ± 2.7	0.73 ± 0.21	(3.5 ± 0.6) × 10^4^	3.2 ± 0.98	0.014 ± 0.008	− 2.7 × 10^12^
	N_2_, *n*‐type	Baseline	7.64 ± 0.85	2.39 ± 0.36	(3.3 ± 0.3) × 10^3^	6.5 ± 0.49	0.083 ± 0.044	− 9.0 × 10^10^
		Env. Control	7.75 ± 0.60	2.53 ± 0.20	(2.0 ± 0.2) × 10^3^	7.2 ± 0.30	0.092 ± 0.079	− 9.0 × 10^10^
		Cycled	7.83 ± 0.80	2.50 ± 0.40	(2.4 ± 0.1) × 10^3^	7.3 ± 0.28	0.090 ± 0.075	− 9.0 × 10^10^
(F_5_PhO)_2_‐F_16_‐SiPc ^a)^	Air, *n*‐type	Baseline	21.6 ± 2.0	0.50 ± 0.22	(4.7 ± 0.8) × 10^4^	5.2 ± 1.8	0.31 ± 0.31	− 1.0 × 10^11^
		Env. Control	28.8 ± 4.1	0.31 ± 0.18	(1.1 ± 0.2) × 10^5^	7.2 ± 2.8	1.0 ± 0.56	− 2.0 × 10^12^
		Cycled	21.8 ± 2.1	0.38 ± 0.10	(2.0 ± 0.2) × 10^5^	5.0 ± 1.0	1.5 ± 0.42	− 5.0 × 10^11^
	N_2_, *n*‐type	Baseline	6.3 ± 4.2	1.64 ± 0.31	(5.6 ± 0.8) × 10^3^	4.7 ± 0.76	0.10 ± 0.07	1.0 × 10^12^
		Env. Control	8.7 ± 3.8	1.92 ± 0.27	(1.0 ± 0.09) × 10^4^	4.2 ± 1.0	0.10 ± 0.07	5.0 × 10^11^
		Cycled	15.1 ± 2.7	1.66 ± 0.28	(1.1 ± 0.08) × 10^4^	5.9 ± 1.3	0.11 ± 0.05	5.0 × 10^11^

^a)^ Bottom gate top contact OTFTs made with Copper phthalocyanine (CuPc), Copper(II) 1,2,3,4,8,9,10,11,15,16,17,18,22,23,24,25‐hexadecafluoro‐29H,31H‐phthalocyanine (F_16_CuPc), silicon bis(pentafluorophenoxy)phthalocyanine (F_10_SiPc) and ((F_5_PhO)_2_‐F_16_‐SiPc) as the semiconductor, characterized as either *p*‐type or *n*‐type (Type) either in Air or N_2_ (Env.). The Test type refers to if the corresponding OTFT was characterized *baseline* (or pristine device), the same device after being continuously cycled as an OTFT for 42–72 h (CuPc = 71.1 h, F_16_CuPc = 41.9 h, F_10_SiPc = 47.8 h and (F_5_PhO)_2_‐F_16_‐SiPc = 48.6 h) labeled as *cycled*, or the control devices under the same environment characterized after 42–72 h labeled *Env. Control*. Each value represents the average with the standard deviation, which the exception of the *I_ON_/I_OFF,_* which uses the standard error. The number of devices tested at each condition is ≈17 except for the baseline measurements, which had *N*≈35, and the *N_int,_* which was extracted from a characteristic device in each parameter;

^b)^ *V_T_* = threshold voltage; *µ* = charge mobility (hole for *p*‐type and electron for *n*‐type) and *I_ON_/I_OFF_* = ratio of on‐current and off‐current; *N*_int_ = interface charge density, SS = Subthreshold slope.

Environmental humidity or oxygen can lead to material degradation or charge trap formation^[^ ^28^, ^29^ ^]^ while operational bias stress can also lead to changes in OTFT performance.^[^ ^19^ ^]^ Oxygen and water in the air can oxidize organic semiconductors, leading to charge trapping and degradation of performance. The oxidation potential of molecular oxygen in air is ≈−5.2 to −5.4 eV. Thus, to avoid oxidation in air for hole transport in *p*‐type OTFTs, the highest occupied molecular orbital (HOMO) level of an organic semiconductor should generally be lower (deeper) than −5.2 eV relative to the vacuum level. If the HOMO level of the organic semiconductor is higher (shallower) than −5.2 eV, oxidation is more likely because the material can donate electrons to oxygen. Oxidation effects on CuPc have been widely studied and have shown a small degradation over long‐term exposure due to its HOMO level of 5.2 eV.^[^ ^20^, ^30^ ^]^

For *n*‐type organic semiconductors, resistance to reduction by oxygen and moisture is critical to maintaining stable OTFT operation in air. To achieve air stability, it has been shown that the lowest unoccupied molecular orbital (LUMO) should be lower (deeper) than ≈−4.0 eV relative to the vacuum level.^[^ ^31^ ^]^ Molecular oxygen has an electron affinity of ≈−4.0 eV, therefore, if the LUMO of the organic semiconductor is higher (shallower) than this threshold, oxygen can easily capture free electrons, leading to unintentional doping and device degradation. Air‐stable *n*‐type materials typically have LUMO levels at or below −4.0 eV, with many stable candidates reaching −4.3 eV or lower, ensuring minimal electron trapping and improved operational stability. This was explored by our group in a previous study, which showed air stability and instability for many silicon phthalocyanines depending on the LUMO level.^[^ ^32^ ^]^ From this study, (F_5_PhO)_2_F_16_SiPc was reported as air stable for *n*‐type performance due to its low LUMO level (≈−4.8 eV) while F_10_SiPc was reported as instable as an *n‐*type OTFT. In a similar study, Anthopoulos et al. showed the stability of F_16_CuPc in air as an *n‐*type, due to its LUMO level (≈−4.5 eV).^[^ ^33^ ^]^ These studies are important to remember when analysing the response, to further isolate potential causes.

**Figure 2:**
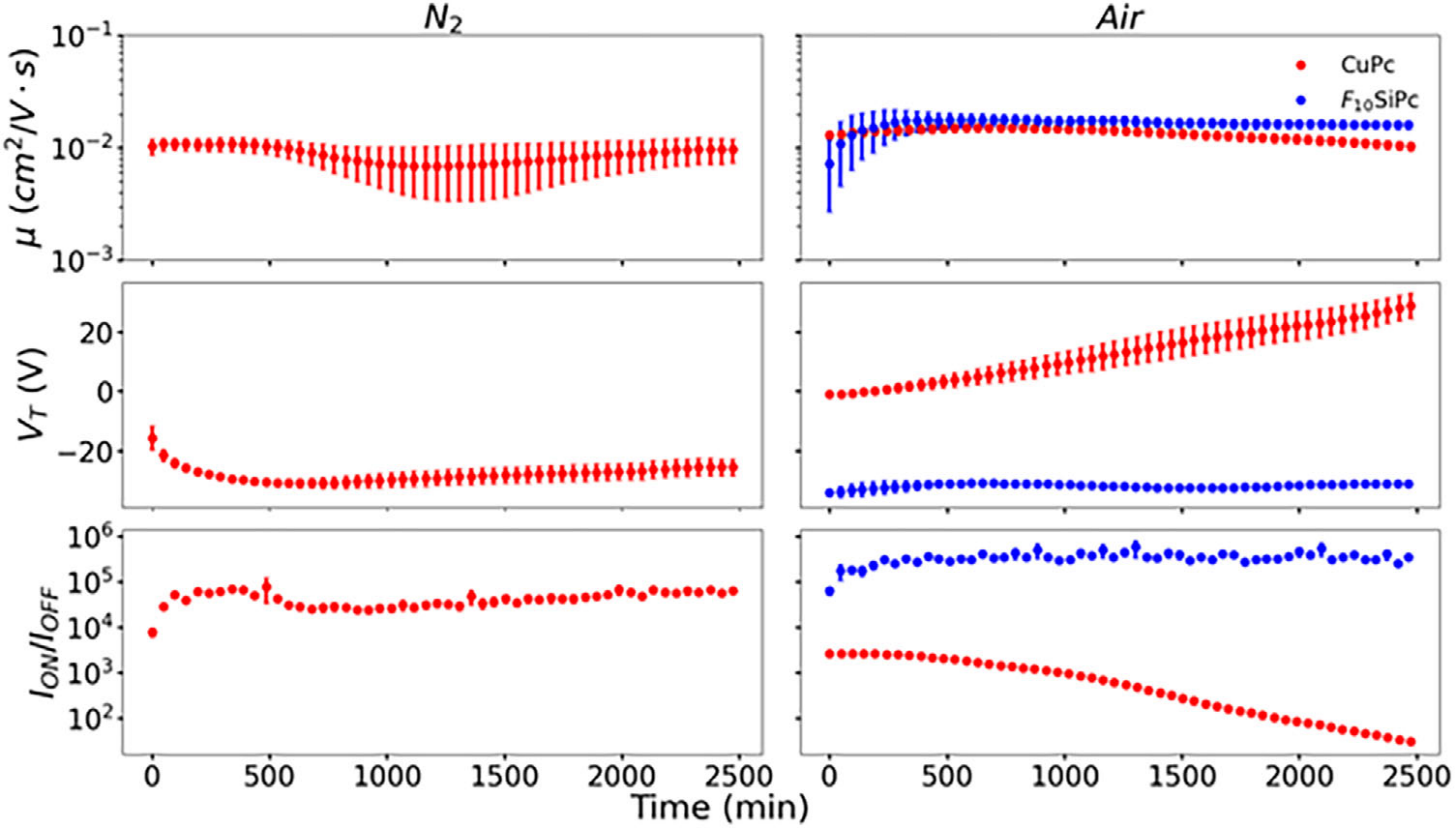
Major parameters (*V*_*T*_, *I*_*ON*_/*I*_*OFF*,_ and *µ*) extracted from *p*‐type OTFTs (CuPc and F_10_SiPc) extracted in air and in N_2_, over time. Each data point represents the average of 15–20 devices with the error bars representing the standard deviation in *V*_*T*_ and *µ*, and the standard error in *I*_*ON*_/*I*_*OFF*_.

**Figure 3:**
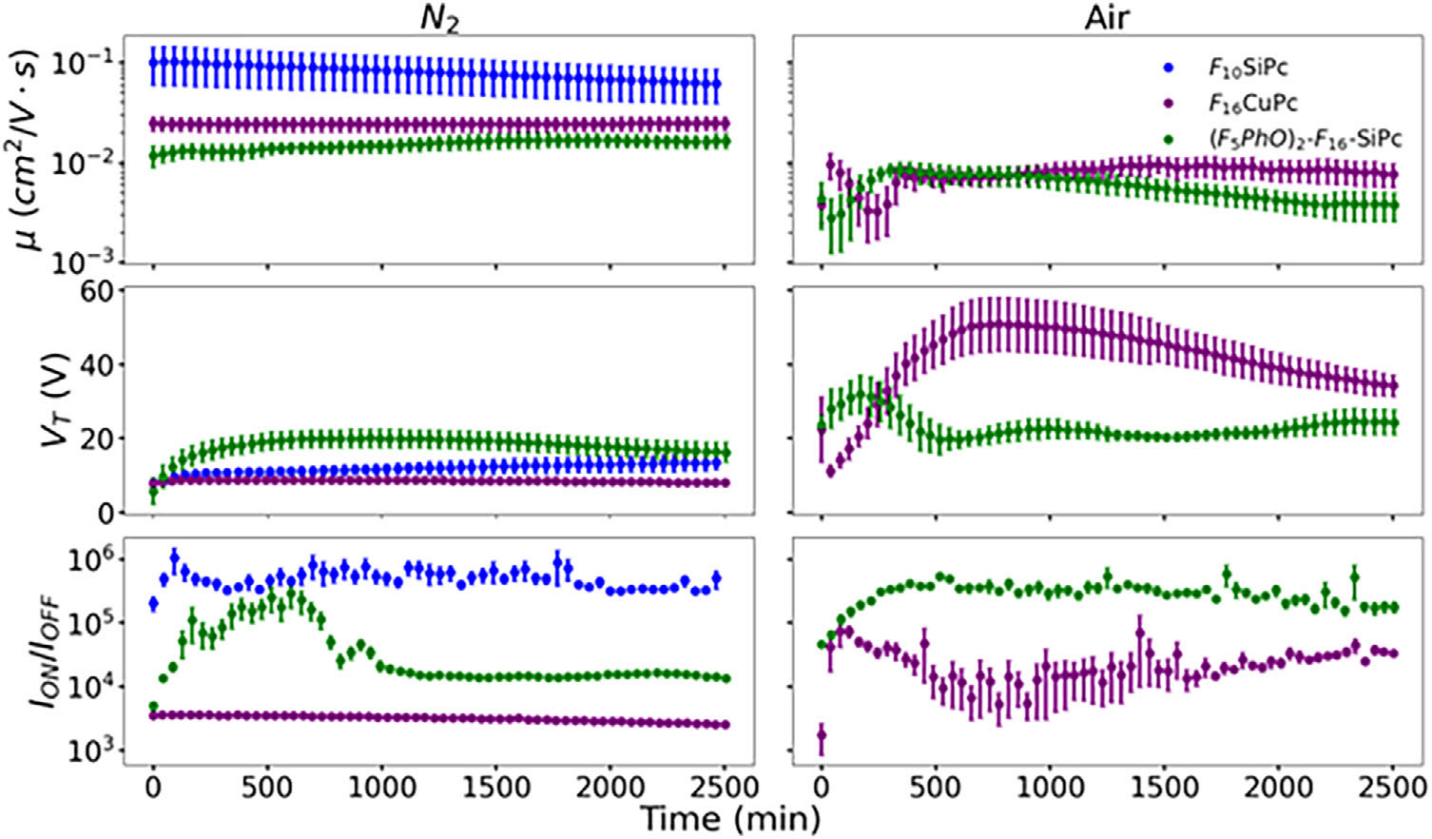
Major parameters (*V*_*T*_, *I*_*ON*_/*I*_*OFF*_, and *µ*) extracted from *n*‐type OTFTs (F_10_SiPc, F_16_CuPc, and F_26_SiPc) in air and in N_2_, over time. Each data point represents the average of 15–20 devices, with the error bars representing the standard deviation in *V*_*T*_ and *µ* and the standard error in *I_ON_*/*I_OFF_*.

To isolate the operational stress effects from environmental effects we characterized 20 devices by taking output curves at set gate‐source voltages (*V_GS_*) and four subsequent transfer curves at the same drain‐source voltage (*V_DS_*) in the saturation regime continuously, one device after the other, for 48–72 h until the device performance stabilized in air and in *N_2_*. Each individual device took ≈ 2 min to characterize, and every 40 min, all 20 devices would be characterized. A set of 20 devices were characterized in air, while the second set of 20 devices (of same device structure) were simultaneously characterized in a glove box, these were the *Cycled* devices. While these 40 devices were being characterized, a second set of 40 devices, 20 in air and 20 in *N_2_* (*Env. Control*), produced using the same manufacturing process and the same environmental conditions were initially characterized once, held for the 48–72 h, and characterized at the end. The reported device performance of the *Baseline* devices is the average of the initial characterization for both the *Cycled* and *Env. Control* in air or *N_2_*. This process was repeated for all four semiconductor types, CuPc, F_10_SiPc, F_16_CuPc, and (F_5_PhO)_2_‐F_16_‐SiPc. Table 1 contains a summary of all initial conditions and final conditions of the OTFT operations, while the gradual change in *I_ON_/I_OFF_*, *V_T_*, and *µ* both in air and *N_2_* are demonstrated for both *p*‐type operation and *n*‐type operation in **Figures** **2** and **3**, respectively. With the subthreshold slope (SS) and hysteresis behavior shown in Figures S1 and S2 (Supporting Information). Plots for each individual organic semiconductor OTFT, displaying the baseline, the cycling, and the environmental control, are shown in Figures S3–S10 (Supporting Information).

### 2.1 Environmental Versus Operational Stability P‐type Operation

As the semiconducting layer is exposed to air, moisture, and oxygen percolate through, resulting in a charge transfer that suppressing electron conduction (*n*‐type) and promoting hole conduction (*p*‐type).^[^ ^28^ ^]^ Therefore, in air, F_10_SiPc only displayed *p*‐type behavior. CuPc is typically regarded as an air‐stable semiconductor but is well known to be susceptible to oxygen and moisture doping.^[^ ^30^ ^]^ We observed similar behaviors for CuPc in air. As the device is cycled, the *V_T_* continuously shifts more positive and the *I_ON_/I_OFF_* ratio decreases until the devices stop working (Figure 2). This is further emphasized when looking at the sub‐threshold sweep, which gradually gets further away from zero (Figure S4, Supporting Information). As the SS increases, so does the density of defects.^[^ ^34^ ^]^ The *Env. Control* device that was kept in air had almost identical device performance as the one cycled in air (Table 1), suggesting this degradation is a result of oxygen and moisture and is not accelerated by bias stress. Surprisingly, their hole mobilities (*µ_h_*) only dropped slightly over the same timeframe. In *N_2_*, the opposite is shown, where the *V_T_* increased (shifted negatively for a *p‐*type semiconductor) with the devices as they were cycled, experiencing a larger shift. *µ_h_* and *I_ON_/I_OFF_* remained mostly constant. This suggests a filling of trap states which, once filled, contribute to the electrostatic charge from the gate voltage, therefore, more voltage is needed to achieve previously obtained carrier concentrations.^[^ ^17^ ^]^ The *Env. Control* devices also showed a slight increase in *V_T_*, but within error of the *Baseline* condition, suggesting the increase in *V_T_* for the cycled device is a result of operation and not environmental factors. Surprisingly, the change in *V_T_* has not affected the hysteresis, which has stayed relatively small. F_10_SiPc is an *n*‐type semiconductor in *N_2_* but when characterized in air it demonstrates good *p‐*type behavior.^[^ ^15^, ^22^, ^23^, ^32^, ^35^, ^36^, ^37^ ^]^ In air, we observe a steady improvement in *µ_h_* and *I_ON_/I_OFF_* with a slight drop in *V_T_* for the first 100 min of operation (Figure 2) before leveling‐off, for the performance of F_10_SiPc based devices. The *Env. Control* also showed improved performance compared to the baseline devices suggesting the F_10_‐SiPc is being oxygen‐doped, leading to improved OTFT performance. F_10_‐SiPc did not demonstrate *p*‐type operation in *N_2_* suggesting the oxygen doping is necessary for *p*‐type operation. Compared to CuPc, F_10_‐SiPc provided a much more air‐stable device overall. Neither F_16_CuPc nor (F_5_PhO)_2_‐F_16_‐SiPc demonstrated significant *p*‐type operation in either air or N_2_.

### 2.2 Environmental Versus Operational Stability N‐type Operation

CuPc did not demonstrate any *n*‐type behavior in either air or *N_2_*, however, F_10_‐SiPc demonstrated good *n*‐type performance in *N_2_* with good stability. We observe statistically similar device performance for baseline devices as the *Env. Control* devices suggesting little material degradation in the glove box. We also observe only a slight increase in *V_T_* and slight drop in electron mobility (*µ_e_*) when cycled in N_2_ for over 48 h (Figure 3; Table 1). F_10_‐SiPc demonstrated negligible *n*‐type behavior in air. F_16_CuPc demonstrated excellent stability in *N_2_* with negligible changes during operation (Figure 3) or for *Env. Control* (Table 1). However, when cycled in air F_16_CuPc experienced significant changes in performance over time and as a function of operation. In the first 100 min, we observe a sharp increase in *µ_e_* and *I_ON_/I_OFF_* with a slight drop in *V_T_*; in the subsequent 1000 min we see an increase in *V_T_* and decrease in *I_ON_/I_OFF_*; finishing off the last 1500 min with a slow return of *V_T_* and increase in *I_ON_/I_OFF_* (Figure 3). The *µ_e_* remained relatively constant after 500 min of operation. The significant difference in *V_T_* between *Env. Control* and *Cycled* suggests the differences are not simply due to air exposure but that applying a bias plays a role (Table 1). (F_5_PhO)_2_‐F_16_‐SiPc has recently been reported as an air‐stable *n*‐type semiconductor in OTFTs with great long‐term environmental stability.^[^ ^38^ ^]^ In *N_2_*, (F_5_PhO)_2_‐F_16_‐SiPc based OTFTs experienced a slight increase in *µ_e_*, *I_ON_/I_OFF_*, and *V_T_* over the first 500–1000 min, after which point the device stabilized and demonstrated constant performance (Figure 3). The *Env. Control* had similar performance to *Baseline* devices while the *Cycled* devices experienced an increase in *V_T_* of roughly 7 V. In air, (F_5_PhO)_2_‐F_16_‐SiPc experienced a stabilization period of roughly 500 min and then demonstrated constant OTFT performance. The *µ_e_*, *I_ON_/I_OFF_*, and *V_T_* for *Baseline*, *Env. Control* and *Cycled* devices were all within statistical error, suggesting very stable device operation in air compared to F_16_CuPc. For each of the devices, the SS and hysteresis remained fairly stable. This suggests that the shift in *V_T_, µ_e_*, and *I_ON_/I_OFF_* in *n*‐type devices may not be directly correlated to the defect density, SS, or the hysteresis.

## 3 Characterizing the Factors Which Influence Instability

### 3.1 Molecular Orientation

**Figure 4:**
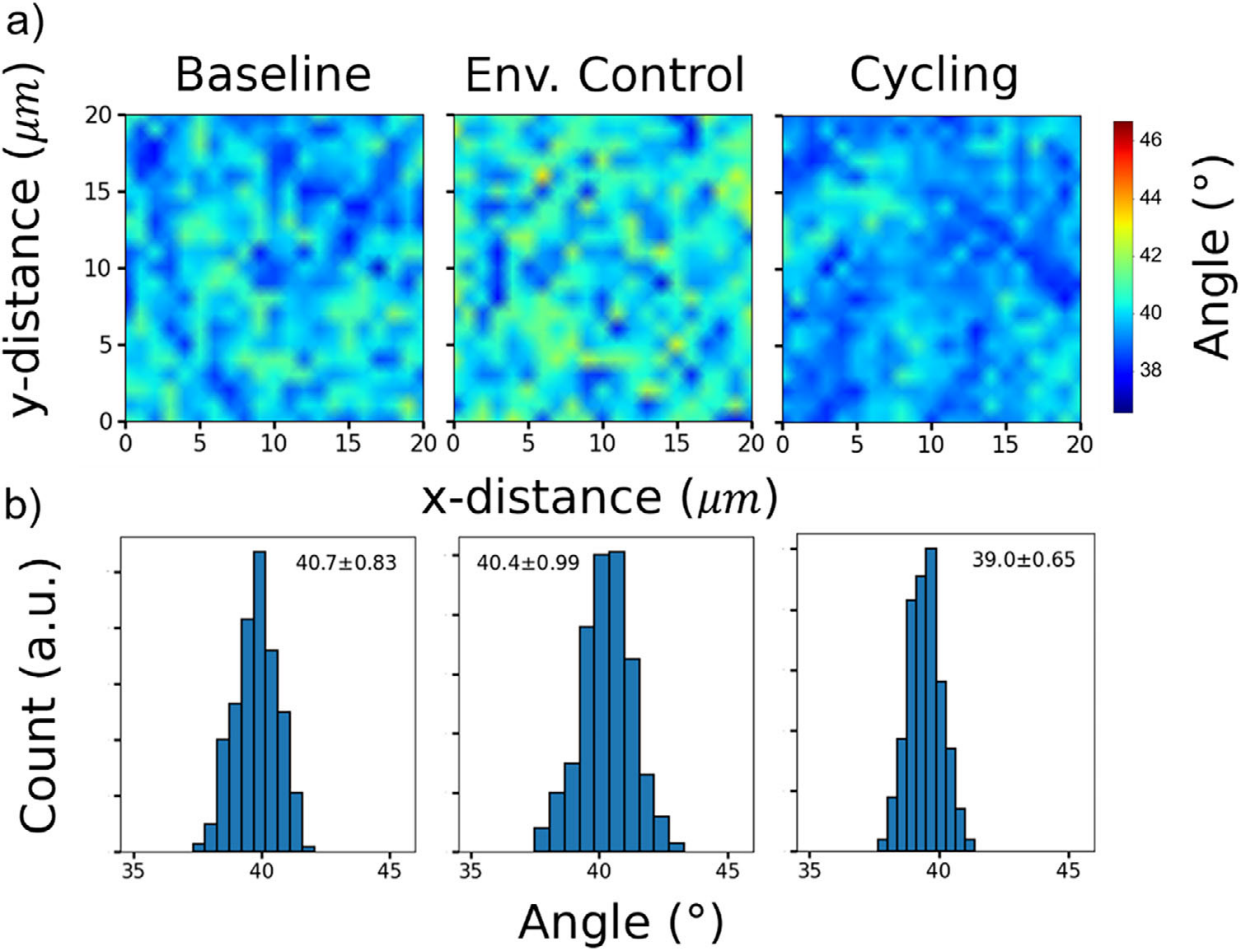
2D plots of the molecular orbital of F_10_SiPc outside the OTFT channel in air for the pristine, environmental, and cycling condition a), with its associated histograms b).

Changes in performance due to environmental or bias stresses are often due to a change in the distribution of charges. These changes in distributions can arise from a change in morphology such as molecular packing or an introduction of impurities which cause doping or from filling trap states.^[^ ^17^ ^]^ Using polarized Raman microscopy, we built maps which identify the average molecular angle of the phthalocyanine to the substrate by calculating the relative intensity of the isoindole peak at ≈1550 cm^−1^ at Z(XX)Z’ and Z(XY)Z’ polarizations.^[^ ^39^, ^40^, ^41^ ^]^ These molecular angle maps provide an overview of the film morphology, which can provide feedback on structural changes as a function of environmental exposure and bias stress. To evaluate the whole OTFT, the polarized Raman microscopy maps were measured inside and outside the OTFT channel, and corresponding histograms showing the distribution of the molecular angles relative to substrate were plotted (Figures S12–S17, Supporting Information). Characteristic polarized Raman microscopy molecular angle maps, along with corresponding histograms for F_10_‐SiPc are reported in **Figure** **4**. For all of the semiconductors, no statistical changes in molecular orientations were observed upon environmental exposure and bias stress. F_10_SiPc, F_16_CuPc, and (F_5_PhO)_2_‐F_16_‐SiPc showed average molecular orientations of 39.9±0.3, 48.7±0.7, and 46.7±0.2 degrees. The values obtained for (F_5_PhO)_2_‐F_16_‐SiPc were consistent with our previous study.^[^ ^42^ ^]^ For the (F_5_PhO)_2_‐F_16_‐SiPc, some of the source and drain electrodes overshadowed the semiconductor, causing discrepancies from either side. This shadowing was not visible via the optical microscope and was only visible after developing the Raman molecular orientation maps. For this reason, the analysed channel was reduced to only capture the semiconductor. (Figures S16 and S17, Supporting Information). Table S1 (Supporting Information) contains the average and standard deviations of these maps for each condition. Since the Raman measurements were performed in air, we could not obtain the molecular orientation for the baseline devices in *N_2_*. Raman spectroscopy of CuPc films showed no changes for multiple points at various locations for all the conditions, therefore we assume no changes in molecular orientation with environmental and bias stress (Figure S11a, Supporting Information). Overall, these results suggest that the observed change in OTFT performance as a function of environmental exposure and/or constant operation is not due to molecular orientation changes in the phthalocyanines films. These changes could stem from electronic structure, chemical stability, and molecular packing, all without noticeably affecting the morphology. For instance, depending on the LUMO and HOMO levels of the organic semiconductor or the reactivity of their functional groups, they may interact with moisture and oxygen in the air, leading to degradation. The morphology also does not inform us on the porosity of the grains, which influence how oxygen and moisture enter the films.

### 3.2 Modeling

Minimal morphological changes in semiconductor films indicate that other factors dominate the observed changes in transfer characteristics. To investigate possible physical and electrical origins, we modeled our devices using the 2D finite‐element method. Organic devices have complex mechanisms. Therefore, parameters extracted from simple methods only reflect partial or non‐contextual elements of device operation.^[^ ^43^, ^44^, ^45^ ^]^Physically based numerical simulation reproduces a whole current–voltage curve in a completely bottom‐up manner, providing an unambiguous set of energetic, transport, and interface parameters.^[^ ^46^, ^47^, ^48^, ^49^, ^50^ ^]^ A major modeling challenge here was to set up a simple yet robust base framework that is applicable to data from four different semiconductors [CuPc, F_16_CuPc, F_10_SiPc, (F_5_PhO)_2_‐F_16_‐SiPc)] and six different test conditions (a full combination of Air/N_2_ and *Baseline*/*Env. Control*/*Cycled*). Based on an iterative process, we selected essential semiconductor physics equations (the Poisson's and drift‐diffusion equations), the Schottky injection model, *N*_int_, and exponential trap density of states (DOS). This framework allowed us to minimize the number of fitting parameters while generating a high‐quality fit to all devices.

**Figure 5:**
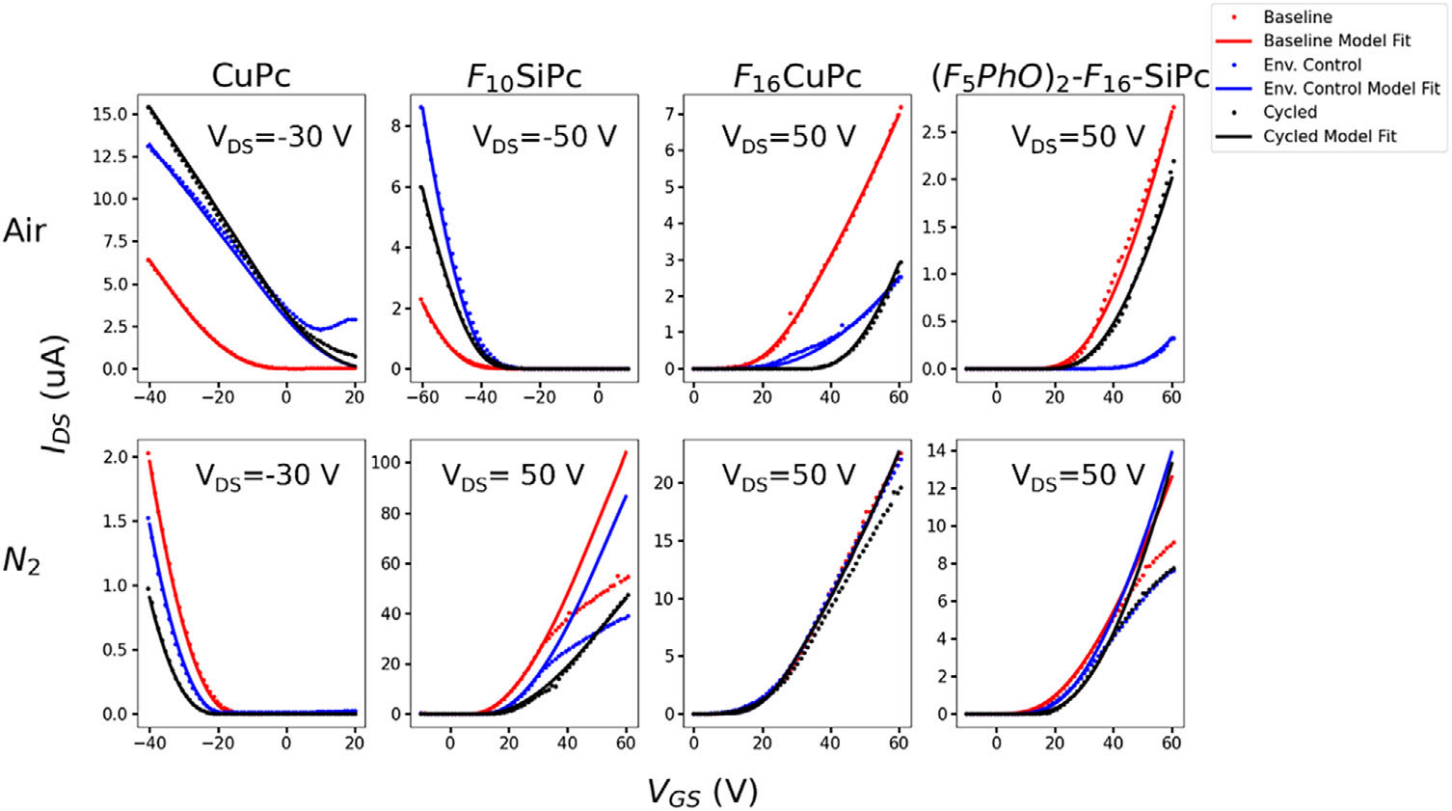
Model fittings on characteristic CuPc, F_10_SiPc, F_16_CuPc, and (F_5_PhO)_2_‐F_16_‐SiPc) device transfer curves left in air, for the pristine, environmental, and the last cycling curves.

The modeling results are shown in **Figure** **5**. A summary of key parameters is shown in Table S2 (Supporting Information). An important observation is that a substantial trap density (*H_D_* or *H_A_*) of the order of 10^18^ cm^−3^ and a high characteristic temperature (*T_CD_* or *T_CA_*) in excess of 1400 K were necessary in all cases to reproduce the major features of transfer characteristics (Table S2, Supporting Information). Considering the polycrystalline nature of evaporated small‐molecule films, these traps are likely to originate from grain boundaries. Interestingly, the two trap parameters of each transistor (*H_D_* and *T_CD_* for a *p*‐channel transistor, *H_A_* and *T_CA_* for an *n*‐channel transistor) remained largely unchanged upon environmental and cycling testing, an argument that is strongly supported by the polarized Raman microscopy data (Figure 4). In contrast, the sign and magnitude of *N*_int_ changed quite dramatically in materials and conditions that led to large *V_T_* shift. The parameter *N*_int_ selectively induces a lateral shift of the transfer curve without its shape change. Therefore, the value of *N*_int_ in each device was determined by scanning its value over an estimated range and identifying the one that produces a simulation curve that shows the best agreement with the experimental curve in terms of *V_T_*. We therefore infer that a fixed 2D charge sheet is formed at the semiconductor/insulator interface upon prolonged exposure to air and/or electrical bias. This charge sheet in turn partially screens the applied gate field producing the observed *V_T_* shift. It should be noted that the trap DOS itself was nonetheless surprisingly resilient to all stress conditions, which can be explained by the morphological stability of materials.

Existence of the contact resistance was considered in our models by specifying and adjusting the electrode work function and semiconductor HOMO and LUMO levels, thus determining the primary charge‐injection barrier of each system (hole barrier for *p*‐type and electron barrier for *n*‐type operation). On the other hand, the bulk resistance of each transistor was controlled and determined by changing its electron and hole mobilities and by optimizing its trap parameters as described above.

Figure 5 and Table 1 show that OTFTs based on F_16_CuPc undergo the most dramatic change between N_2_ and air conditions; exceptionally stable in N_2_, while its environmental and cycling stability decreased rapidly when exposed to air. As the statistical results in Figure 3 and Table 1 involve measuring 20 different transistors under each condition, the degree of *N_int_* variation and the corresponding V_T_ shift in air demonstrated a greater scatter for F_16_CuPc compared to other materials.

The deviation of the experimental curves of the F_10_SiPc and (F_5_PhO)_2_‐F_16_‐SiPc transistors in N_2_ from the modelling curves suggests injection‐limited behaviors at high *V*_GS_, which is likely to be a common characteristic of SiPc based *n*‐type OTFTs. As a minimal change in the transfer curve was observed in the F_16_CuPc transistor in N_2_, we provided a single modelling curve that describes global characteristics of three experimental curves for that device in Figure 5.

## 4 Conclusion

OTFT stability characterization was performed on four semiconducting materials: CuPc, F_16_CuPc, F_10_SiPc, (F_5_PhO)_2_‐F_16_‐SiPc) in a bottom‐gate, top‐contact geometry, in air and *N_2_*. Our autotester enabled consecutive testing of 20 OTFTs for 48–72 h providing statistically significant cycling characteristics of the OTFTs using different materials. We demonstrate that seemingly small changes in molecular structure have significant effects on the devices’ response to cycling in different environments. F_10_‐SiPc provided a much more air‐stable *p‐*type operation compared to CuPc. In *N_2_*, (F_5_PhO)_2_‐F_16_‐SiPc based OTFTs experienced slight increase in *µ_e_*, *I_ON_/I_OFF_* and *V_T_* over the first 500–1000 min, where then the device stabilized and demonstrated constant performance while F_16_CuPc showed very stable *n*‐type performance in *N_2_*. In air, (F_5_PhO)_2_‐F_16_‐SiPc experienced stable *n*‐type device operation in air compared to F_16_CuPc which experienced significant degradation with cycling. While all materials appear comparable with initial operation, continuous cycle operation leads to different performance changes which is important for circuit design and applications considerations. We performed Raman microscopy on the films before and after stress testing and concluded that no significant change in morphology or chemical signature (changes in chemical bonds) was observed suggesting the change in performance was not structural. Using the 2D finite‐element method, the main cause for the changes in device performance was correlated to the changes in *N*_*int*_ fixed at the semiconductor/insulator interface. This study demonstrates the use of high throughput OTFT characterization for stress testing for extended periods of time providing insight into performance stability and structure property relationships that govern them.

## 5 Experimental Section

### Materials

Copper phthalocyanine (CuPc, 90%, TCI P100525G) and copper(II) 1,2,3,4,8,9,10,11,15,16,17,18,22,23,24,25‐hexadecafluoro‐29H,31H‐phthalocyanine (F_16_‐CuPc, >98%, TCI H1194) were purchased from TCI Chemicals and further purified using train sublimation. Bis(pentafluorophenoxy) hexadecafluoro silicon(iv) phthalocyanine ((F_5_PhO)_2_‐F_16_‐SiPc), and silicon bis(pentafluorophenoxy) phthalocyanine (F_10_SiPc) were synthesized as previously described by our group.^[^ ^15^, ^42^ ^]^ Trichloro(octyl)silane (OTS, 97%) was purchased from Sigma Aldrich and was used as received. All other chemicals were used as received unless otherwise specified.

### Fabrication

N‐doped Si substrates with a 230 nm thick dielectric layer of SiO_2_ were purchased from Ossila and diced into 15 × 20 mm rectangles. Before fabrication, substrates were sequentially sonicated in deionized water, acetone, and isopropanol for 5 min each, using a VWR ultrasonic cleaner (Model No: 97043–964), followed by drying using a constant flow of dry nitrogen. Substrates were then plasma cleaned for 15 min using air‐plasma in a Harrick Plasma PDC‐32G. Plasma‐cleaned substrates were then rinsed with deionized water and isopropanol before being dried with nitrogen. Once dry, substrates were submerged in a solution of 1% v/v octyl‐(trichloro) silane (OTS) in toluene, for 1 h at 70 °C. These served as the gate and dielectric layer for the fabrication of BGTC TFTs. The OTS‐treated substrates were transferred to nitrogen filled glovebox before being fitted with shadow masks and loaded into an Angstrom Engineering EvoVac thermal evaporator used for physical vapor deposition of the desired semiconductors (CuPc, F_10_SiPc, F_16_CuPc or (F_5_PhO)_2_‐F_16_‐SiPc). All semiconductors were deposited on a heated substrate maintained at 140 °C and at a pressure below 2 × 10^−6 ^Torr at a rate of 0.2 Å s^−1^ until reaching a maximum thickness of 300 Å for the CuPc, 500 Å for the F_10_SiPc, 400 Å for the F_16_CuPc and 500 Å for the (F_5_PhO)_2_‐F_16_‐SiPc but unheated, these were chosen from past studies with good performance.^[^ ^22^, ^27^, ^38^, ^40^, ^41^ ^]^ During evaporation, the substrates were rotated at 10 RPM to ensure uniform coverage. The substrates were then cooled to ambient temperature for 24 h. A diamond‐tipped pen was used to expose the Si base by scratching the corners of the substrate, allowing deposition of the gate electrode. Electrodes were patterned using an Ossila source‐drain shadow mask that determines the channel length (*L*) of 30 µm and the channel width (*W*) of 1000 µm. In the same evaporator, the substrates were placed for the electrode deposition: a 500 Å thick layer of 99.99% Au, deposited at 0.5 Å s^−1^ for the CuPc and F_16_CuPc, 100 Å of 99.95% Mn followed by 500 Å of 99.99% Ag for F_10_SiPc and 500 Å of 99.99% Ag for (F_5_PhO)_2_‐F_16_‐SiPc, purchased from Angstrom Engineering. The electrode thicknesses and types were chosen to improve work function alignment and adhesion between the semiconductor and electrodes. Each substrate was patterned with 20 OTFTs with two common gate electrodes. Each semiconductor type was evaporated on four substrates each, two for nitrogen testing and two for air testing.

### Electrical Characterization

Electrical characterization of the OTFTs was performed by analyzing the transfer and the output curves. The chips were all tested using a custom Autotester accompanied by a custom LabVIEW program interfacing with a Keithley 2614B, which has the ability to switch between which OTFT to test and to run output and transfer curves automatically and independently.^[^ ^27^ ^]^ *µ*, and *V_T_* were calculated from the current in the saturation region of the transfer curve:

(1) $$I_{DS}=\frac{\mu WC_{i}}{2L}{\left(V_{GS}-V_{T}\right)}^{2}$$

where *C_i_* is the capacitance density of SiO_2_. By rearranging this, the mobility is obtained, linearly related to the slope of the $\sqrt{I_{DS}}$ versus *V_GS_*.

(2) $$\mu =\frac{2L}{WC_{i}}{\left(\frac{\partial \sqrt{I_{DS}}}{\partial V_{GS}}\right)}^{2}$$

With *V_T_* being x‐intercept of the linear fit used to calculate *µ*. To calculate the subthreshold slope, the slope of the logarithmic transfer curve was taken before the threshold voltage. The hysteresis was simply taken from the absolute difference between the threshold voltage from the forward and reverse transfer curve sweep.

For CuPc, the output curves were run with a *V_DS_* between 20 and −60 V with *V_GS_* set from 20 to −40 V for 7 intervals. For each device, 4 successive transfer curves were run with each voltage point pulsed, ranging from 20 to −40 V with *V_DS_* set at −30 V. For F_10_SiPc in air, the output curves were run with a *V_DS_* between 0 and −50 V with *V_GS_* set from 10 to −60 V at for 6 intervals. The transfer curves were run with each voltage point pulsed, ranging from 10 to −60 V with *V_DS_* set at −50 V. F_10_SiPc in N_2_ had the same ranges as in air but with the signs changed. With F_16_CuPc, the output curves were run with a *V_DS_* between 0 and 60 V with *V_GS_* set from 0 to 60 V at for 5 intervals. The transfer curves were run ranging from 0 to 60 V with *V_DS_* set at 50 V. Finally, the devices with (F_5_PhO)_2_‐F_16_‐SiPc were characterized with output curves with a *V_DS_* between 0 and 60 V with *V_GS_* set from −10 to 60 V at for 5 intervals with transfer curves ranging from −10 to 60 V with *V_DS_* set at 50 V. Each of these ranges were selected to properly characterize the devices throughout the entire testing period.

### Statistical Analysis

All reported OTFT values represent the average of 20 independently fabricated devices (*n* = 20). Data were assessed for consistency, and no transformation, normalization, or outlier removal was applied (only non‐functioning devices were removed). Results were presented as mean ± standard deviation (SD). Time‐dependent behavior was evaluated by plotting the average value of the 20 devices at each time point. No formal hypothesis testing was performed. All data analysis and plotting were conducted using Matlab.

### Raman Characterization

A Renishaw inVia Quontor confocal Raman microscope with a Leica Microsystems brightfield microscope, containing a lightsource DM2700, was used to take polarized and unpolarized Raman microscopy of each organic semiconductor type. Before each measurement, the microscope was calibrated using the silicon's 520 cm^−1^ peak, ensuring accuracy within 0.5 cm^−1^. Both Raman measurements, polarized (Z(X,X)Z′ and Z(X,Y)Z′) and unpolarized, used a 532 nm laser at 0.5 W with a 2400 l mm^−1^ grating to collect spectra from 550 to 1700 mm^−1^. The polarized Raman maps were done at 5% laser power for 2 s/spot, taken every 1 µm over an area of 20 µm × 20 µm except for measurements, which were constrained by the size of the channel, these were 19 µm × 19 µm, all at a magnification of X50L. The non polarized single spectras were done at 10% for 2 s at × 50L magnification. The objective and laser combination has a theoretical spactial resolution and depth of focus of 640 nm and 3.0 µm respectively with a spectral resolution full width half mast (FWHM) of 0.3 cm^−1^. Using the intensity of the pyrrole stretch peak at 1555 cm^−1^ for F_10_SiPc and (F_5_PhO)_2_‐F_16_‐SiPc and 1533 cm^−1^ for F_16_CuPc, for each polarization (I_xx_ and I_xy_) extracted using Wire 5.6 inVia software, the angle of the planar, α, was obtained:^[^ ^51^, ^52^, ^53^, ^54^ ^]^

(3) $$I_{\mathrm{XX}}/I_{\mathrm{XY}}=2\;\mathrm{co}t^{2}\;\alpha$$

A python code was then used to calculate the angle and plot it on a 2D color plot with a Gaussian blur.

### Modeling Methods

A physically based 2D finite‐element numerical solver was used for the simulation of OTFTs (ATLAS, Silvaco). The Poisson's equation was solved on a 2D coordinate system to relate the potential variation to the space charge density,

(4) $$\mathrm{div}\left(\varepsilon_{s}\nabla \varphi \right)=-\rho$$

where *φ* is the electrostatic potential, *ε_s_* is the semiconductor permittivity, and *ρ* is the space charge density. The electrical currents are modelled by drift‐diffusion mechanism,

(5) $$\vec{J_{e}}=qn\mu_{e}\vec{E}+qD_{e}\nabla n$$

and

(6) $$\vec{J_{h}}=qp\mu_{h}\vec{E}-qD_{h}\nabla p$$

where $\vec{J_{e}}$ and $\vec{J_{h}}$ are the electron and hole current density vectors, respectively, $\vec{E}$ is the electric field vector, *µ_e_* and *µ_h_* are the electron and hole mobility, respectively, *D_e_* and *D_h_* are the electron and hole diffusion coefficient, respectively, *q* is the elementary charge, *n* and *p* are the electron and hole concentration, respectively. For OTFTs showing *p*‐type behavior, a donor‐like exponential trap DOS was added to the HOMO edge. The functional form of this DOS is

(7) $$N_{D}\left(E\right)=\frac{H_{D}}{kT_{\mathrm{CD}}}\exp\left(-\frac{E-E_{v}}{kT_{\mathrm{CD}}}\right)$$

where *E* is the electron energy, *H*_D_ is the total density of donor‐like trap states, *k* is the Boltzmann constant, *T*_CD_ is the characteristic temperature of donor‐like traps, and *E_v_* is the energy of the valence band (or HOMO) edge. For OTFTs showing *n*‐type behavior, an acceptor‐like exponential trap DOS was added to the LUMO edge. The functional form of this DOS is
(8)

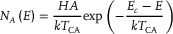

where *H*_A_ is the total density of acceptor‐like trap states, *T*_CA_ is the characteristic temperature of acceptor‐like traps, and *E*_c_ is the energy of the conduction band (or LUMO) edge. The simulator self‐consistently solves Equations (4)–(6) and Equation (7) or (8) over a 2D mesh that is defined to mimic the cross‐section of a fabricated device.

## Conflict of Interest

The authors declare no conflict of interest.

## Supporting information



Supporting Information

## Data Availability

The data that support the findings of this study are available from the corresponding author upon reasonable request.
