# Supplementary material for: Operational and Environmental Stability Assessment of Silicon and Copper Phthalocyanine‐Based OTFTs
Source: Small Methods. 2025 Aug 15;9(9):e00782. doi: 10.1002/smtd.202500782 (PMC12464811; doi:10.1002/smtd.202500782)
Supplement: Supplementary file 1 — Supporting Information [file SMTD-9-e00782-s001.docx]

**Electronic Supporting Information**

Operational and Environmental Stability Assessment of Silicon and Copper Phthalocyanine-Based OTFTs

*Nicholas Dallaire^1^, Joonhyung Park^1,2^, Raluchukwu B. Ewenike^3^, Halynne R. Lamontagne^3,^,* *Chang-Hyun Kim^1^ and Benoît H. Lessard^1,3^**

1: School of Electrical Engineering and Computer Science, University of Ottawa, 800 King Edward Ave., Ottawa, ON K1N6N5, Canada

2: School of Electronic Engineering, Gachon University, Seongnam 13120, Republic of Korea

3: Department of Chemical and Biological Engineering, University of Ottawa, 161 Louis Pasteur, Ottawa, ON K1N6N5, Canada

*Corresponding Author. E-mail: [benoit.lessard@uottawa.ca](mailto:benoit.lessard@uottawa.ca) (BHL)

*
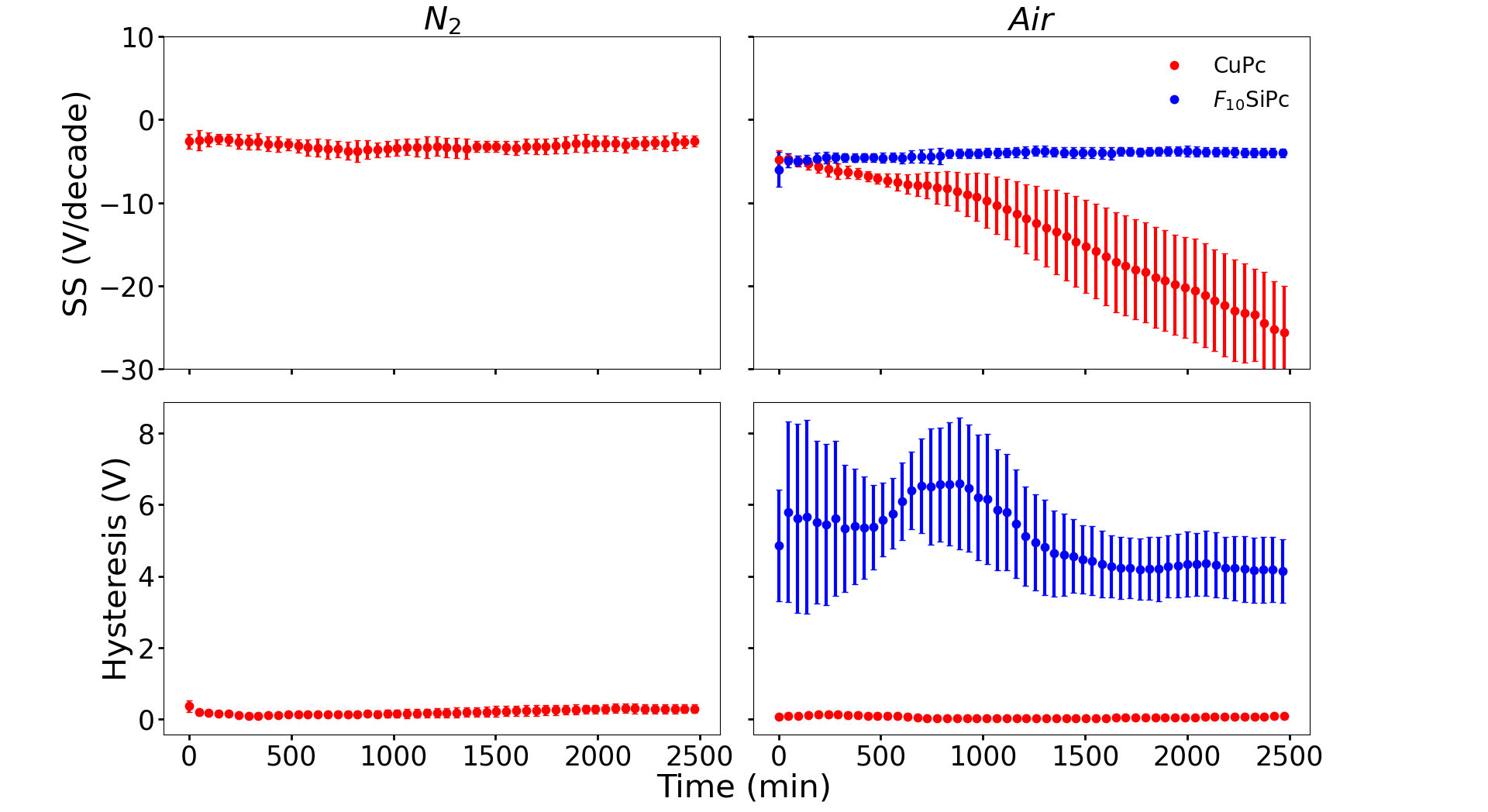
*

**Figure S1)** Sub-Threshold Swing slope, SS and the Hysteresis extracted from p-type OTFTs (CuPc and F_10_SiPc) extracted in air and in N_2_, over time. Each data point represents the average of 15-20 devices with the error bars representing the standard deviation.


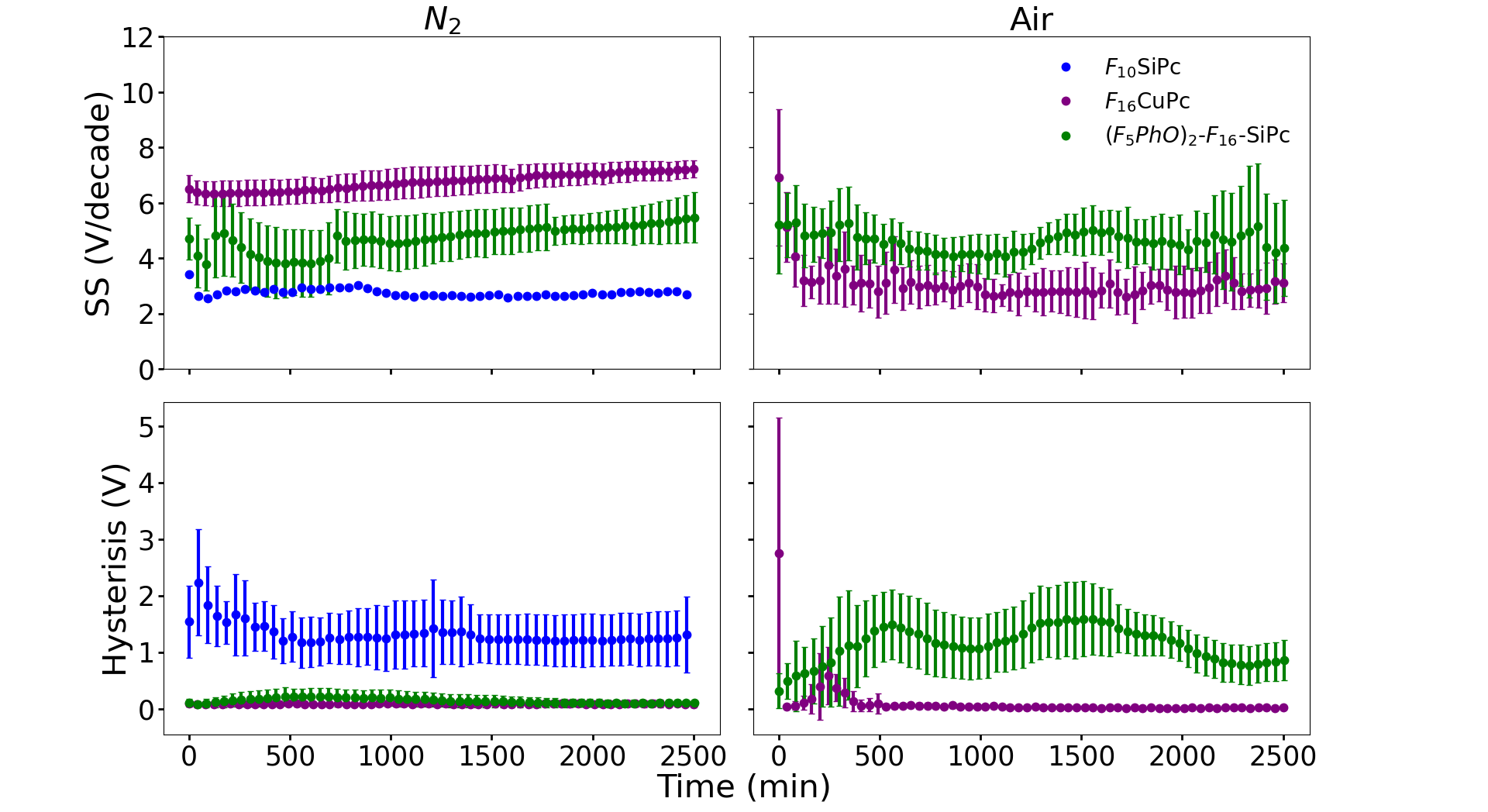
**Figure S2)** Major parameters (SS and Hysteresis) extracted from n-type OTFTs (F_10_SiPc, F_16_CuPc and F_26_SiPc) in air and in N_2_, over time. Each data point represents the average of 15-20 devices with the error bars representing the standard deviation.


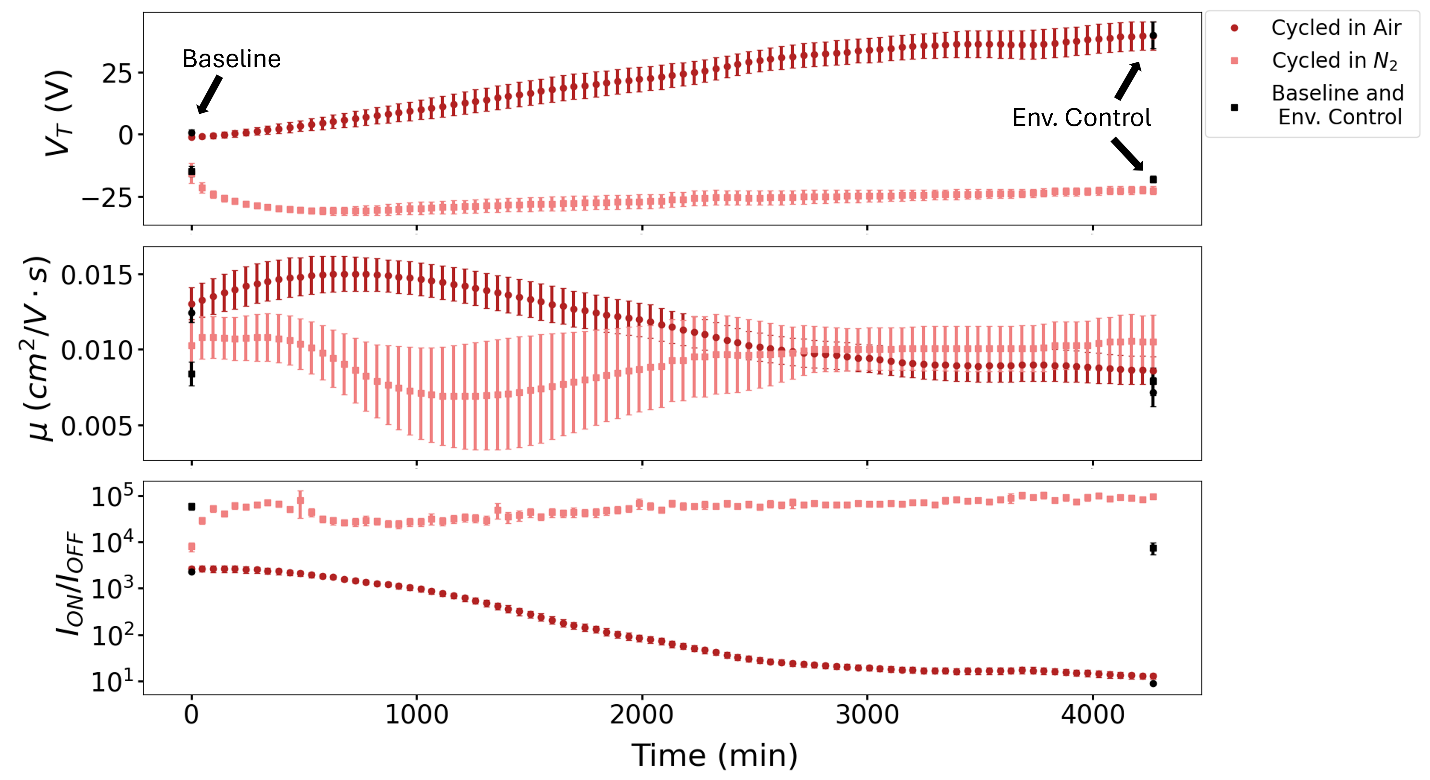


**Figure S3)** Major parameters (V_T_, I_ON_/I_OFF_ and µ) extracted from p-type OTFT CuPc in air and in N_2_, over time. Each data point represents the average of 15-20 devices (except for the baseline, which represents 30-40 devices) with the error bars representing the standard deviation in V_T_ and µ and the standard error in I_ON_/I_OFF_.


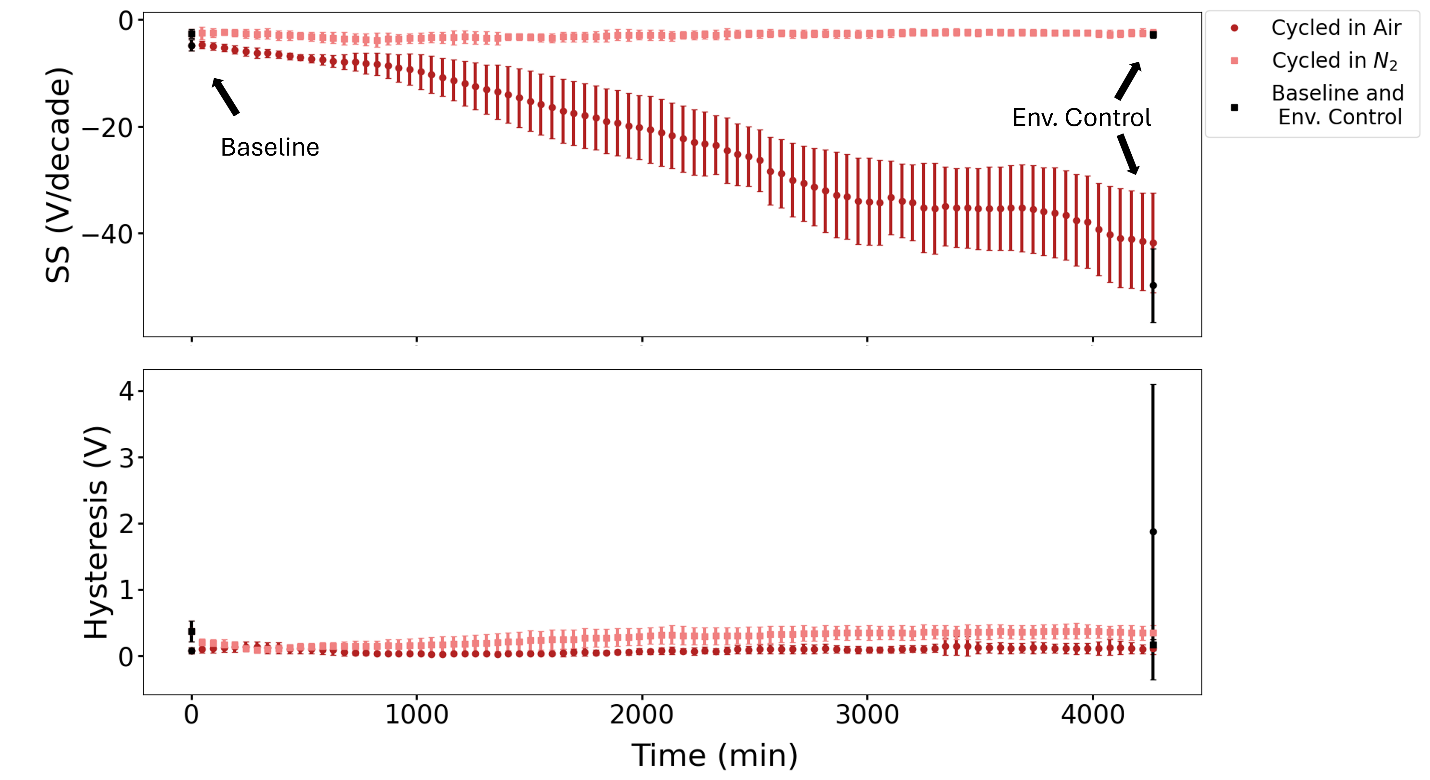


**Figure S4)** Major parameters (SS and Hysteresis) extracted from p-type OTFT CuPc in air and in N_2_, over time. Each data point represents the average of 15-20 devices (except for the baseline, which represents 30-40 devices) with the error bars representing the standard deviation.


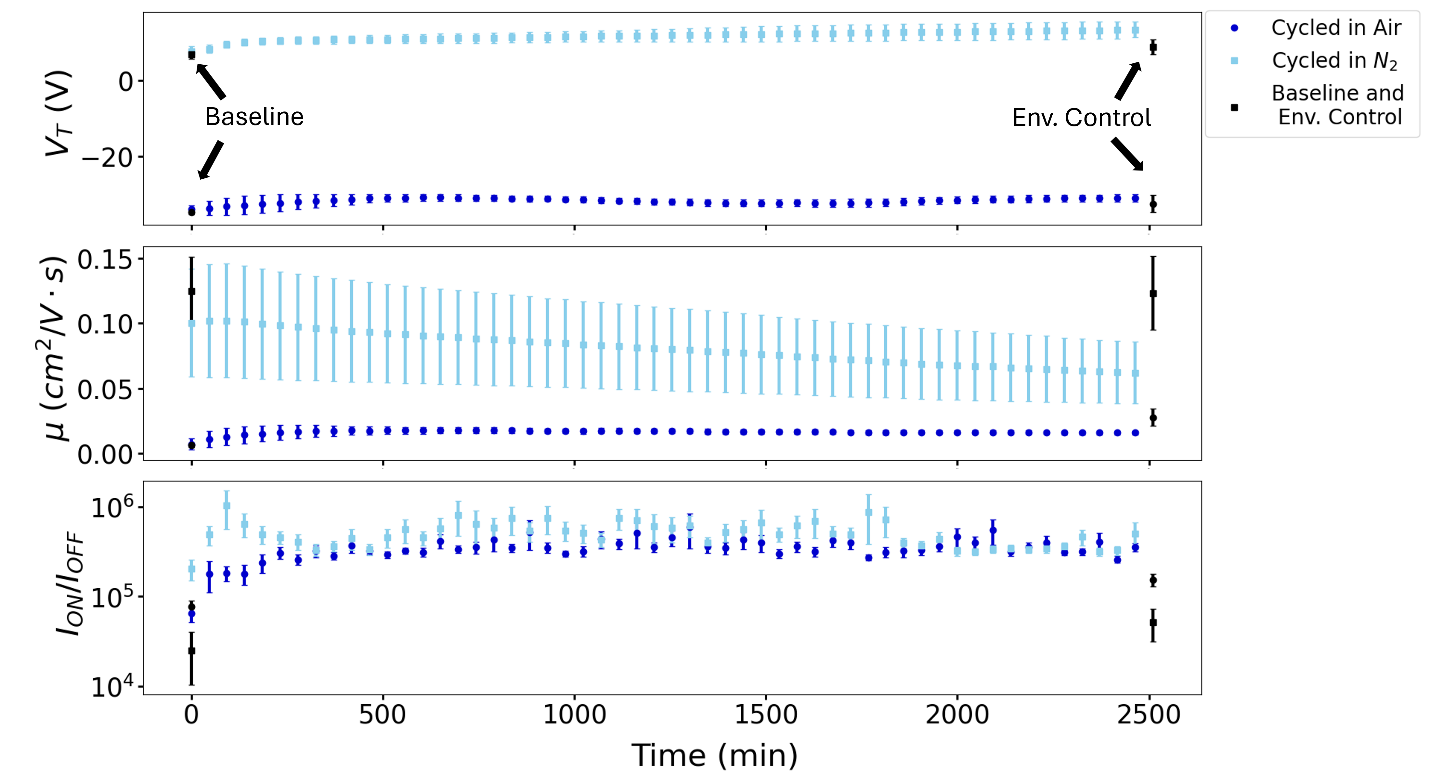


**Figure S5)** Major parameters (V_T_, I_ON_/I_OFF_ and µ) extracted from p-type in air and n-type in N_2_, OTFT F_10_SiPc over time. Each data point represents the average of 15-20 devices (except for the baseline, which represents 30-40 devices) with the error bars representing the standard deviation in V_T_ and µ and the standard error in I_ON_/I_OFF_.


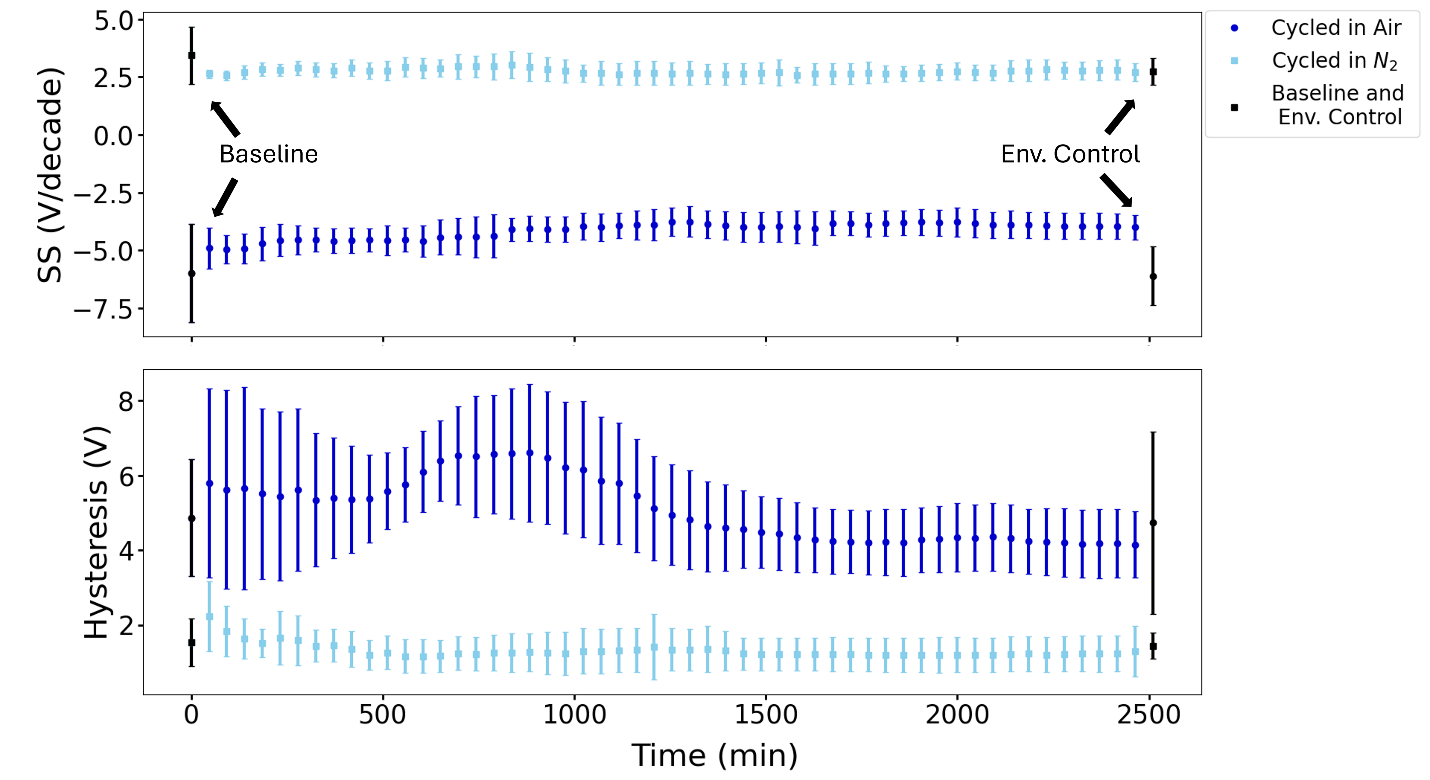


**Figure S6)** Major parameters (SS and Hysteresis) extracted from p-type in air and n-type in N_2_, OTFT F_10_SiPc over time. Each data point represents the average of 15-20 devices (except for the baseline, which represents 30-40 devices) with the error bars representing the standard deviation.


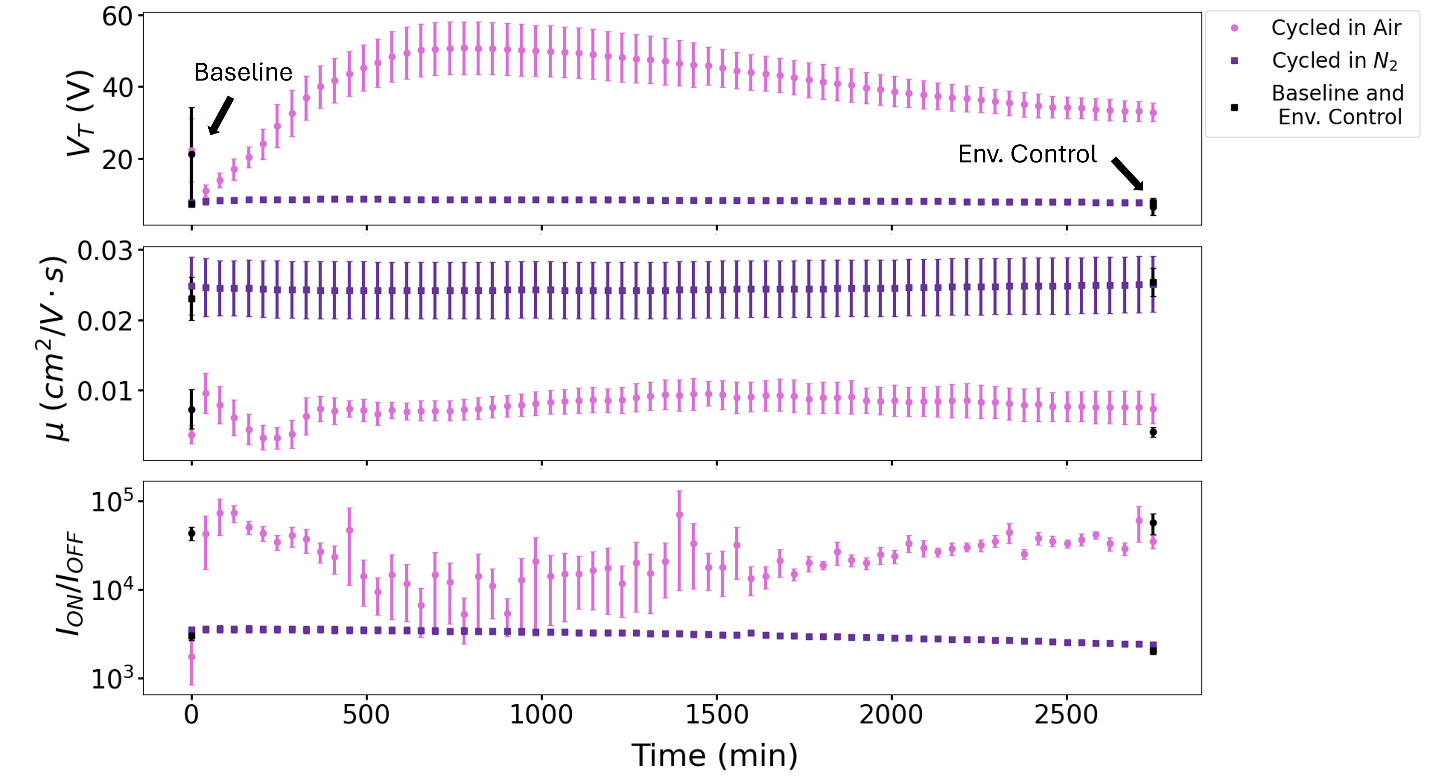


**Figure S7)** Major parameters (V_T_, I_ON_/I_OFF_ and µ) extracted from n-type OTFT F_16_CuPc in air and n-type in N_2_, over time. Each data point represents the average of 15-20 devices (except for the baseline, which represents 30-40 devices) with the error bars representing the standard deviation in V_T_ and µ and the standard error in I_ON_/I_OFF_.


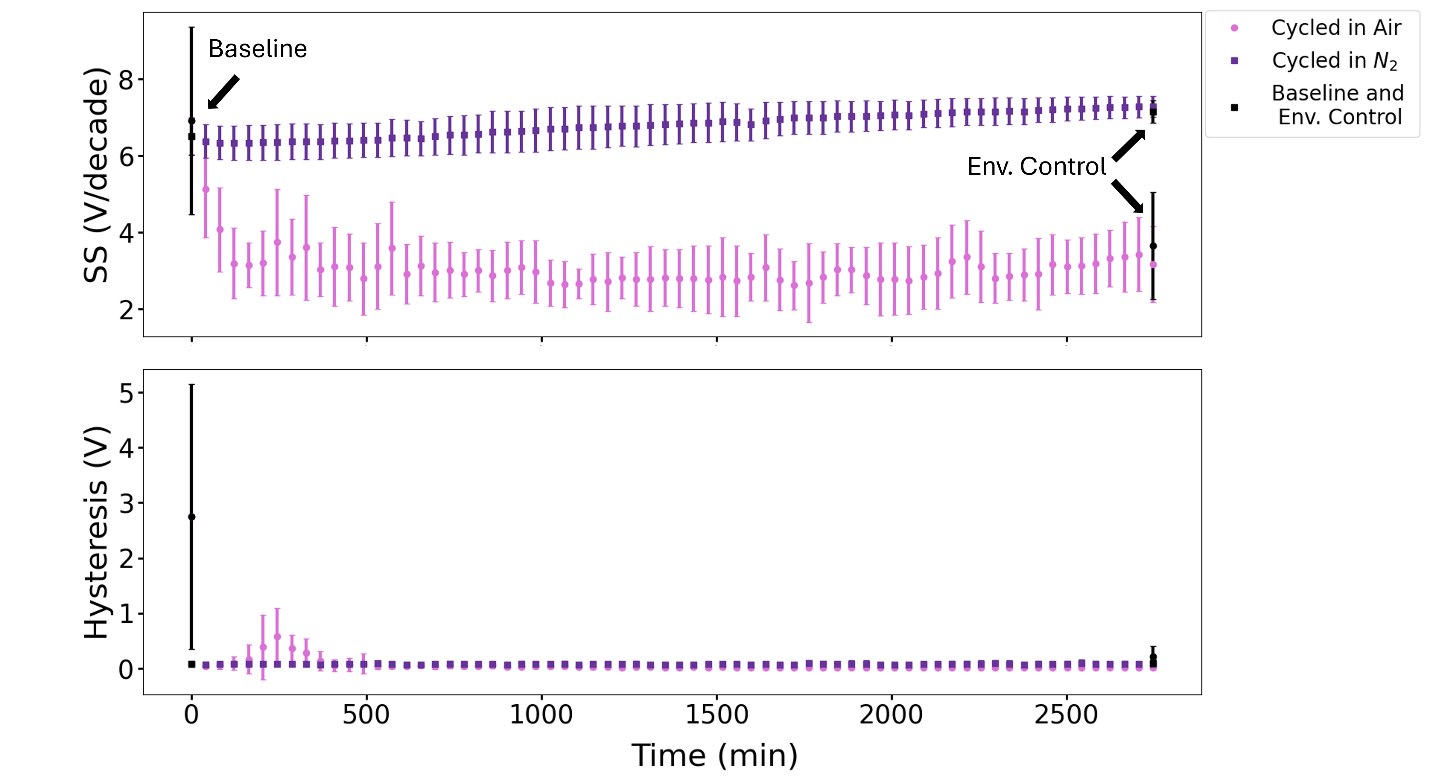


**Figure S8)** Major parameters (SS and Hysteresis) extracted from n-type OTFT F_16_CuPc in air and n-type in N_2_, over time. Each data point represents the average of 15-20 devices (except for the baseline, which represents 30-40 devices) with the error bars representing the standard deviation.


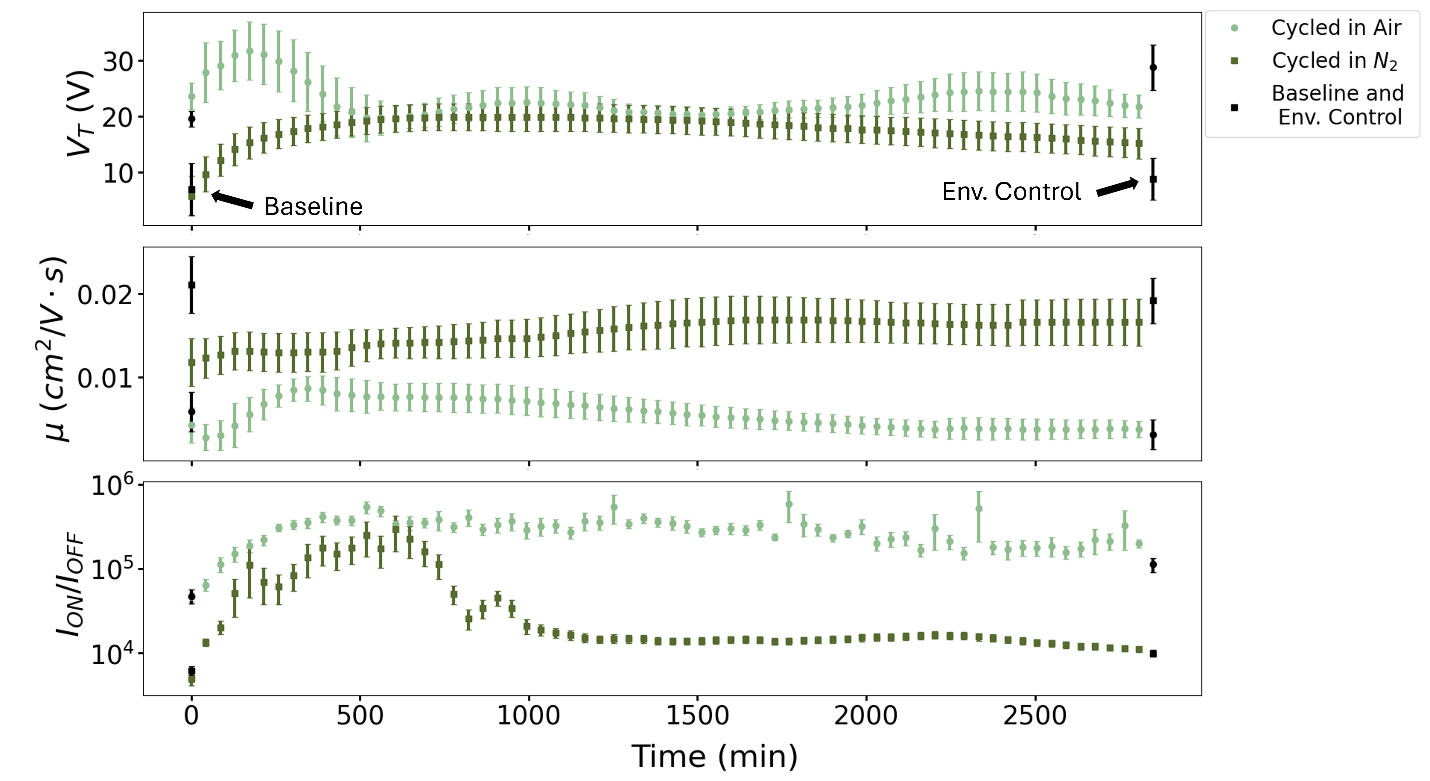


**Figure S9)** Major parameters (V_T_, I_ON_/I_OFF_ and µ) extracted from n-type OTFT (F_5_PhO)_2_-F_16_-SiPc in air and n-type in N_2_, over time. Each data point represents the average of 15-20 devices (except for the baseline, which represents 30-40 devices) with the error bars representing the standard deviation in V_T_ and µ and the standard error in I_ON_/I_OFF_.


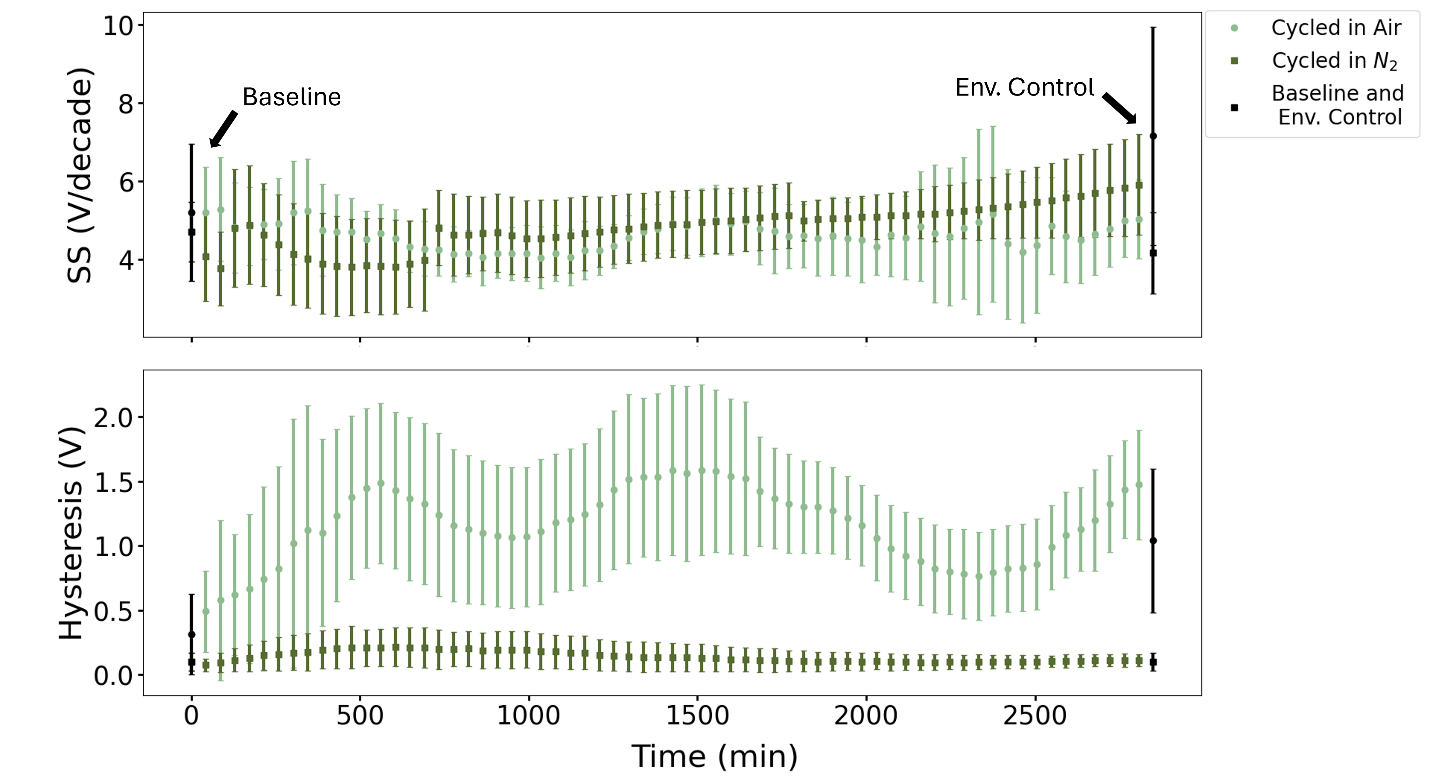


**Figure S10)** Major parameters (SS and Hysteresis) extracted from n-type OTFT (F_5_PhO)_2_-F_16_-SiPc in air and n-type in N_2_, over time. Each data point represents the average of 15-20 devices (except for the baseline, which represents 30-40 devices) with the error bars representing the standard deviation.

**Table S1**) The semiconductor’s average surface molecular orientation in each condition with its standard deviation, extracted via polarized Raman microscopy.

|  | Env. ^a)^ | Type ^a)^ | Test type ^a)^ | Molecular Orientation  (Degrees)  ^b)^ |
| --- | --- | --- | --- | --- |
| F_10_SiPc ^a)^ | Air | p-type | Baseline | 39.79±0.83 |
|  |  |  | Env. Control | 40.29±0.99 |
|  |  |  | Cycled | 39.46±0.65 |
|  | N2 | n-type | Baseline | - |
|  |  |  | Env. Control | 39.84±0.86 |
|  |  |  | Cycled | 40.19±0.81 |
| F_16_CuPc ^a)^ | Air | n-type | Baseline | 47.5±1.0 |
|  |  |  | Env. Control | 49.10±0.90 |
|  |  |  | Cycled | 48.96±0.77 |
|  | N2 | n-type | Baseline | - |
|  |  |  | Env. Control | 49.07±0.84 |
|  |  |  | Cycled | 48.86±0.90 |
| (F_5_PhO)_2_-F_16_-SiPc  ^a)^ | Air | n-type | Baseline | 46.66±0.40 |
|  |  |  | Env. Control | 46.80±0.42 |
|  |  |  | Cycled | 46.94±0.44 |
|  | N2 | n-type | Baseline | - |
|  |  |  | Env. Control | 46.37±0.40 |
|  |  |  | Cycled | 46.77±0.39 |

^a)^ Bottom gate top contact OTFTs made with Copper(II) 1,2,3,4,8,9,10,11,15,16,17,18,22,23,24,25-hexadecafluoro-29H,31H-phthalocyanine (F_16_CuPc), silicon bis(pentafluorophenoxy)phthalocyanine (F_10_SiPc) and ((F_5_PhO)_2_-F_16_-SiPc) as the semiconductor, characterized as either p-type or n-type (Type) either in Air or N_2_ (Env.). The Test type refers to if the corresponding OTFT was characterized *baseline* (or pristine device), the same device after being continuously cycled as an OTFT for 42-72 h (F_16_CuPc = 41.9 h, F_10_SiPc = 47.8 h and (F_5_PhO)_2_-F_16_-SiPc = 48.6 h) labeled as *cycled*, or the control devices under the same environment characterized after 42-72 h labeled *Env. control*.

^b)^ Molecular orientation of crystalline film using polarized Raman microscopy

*
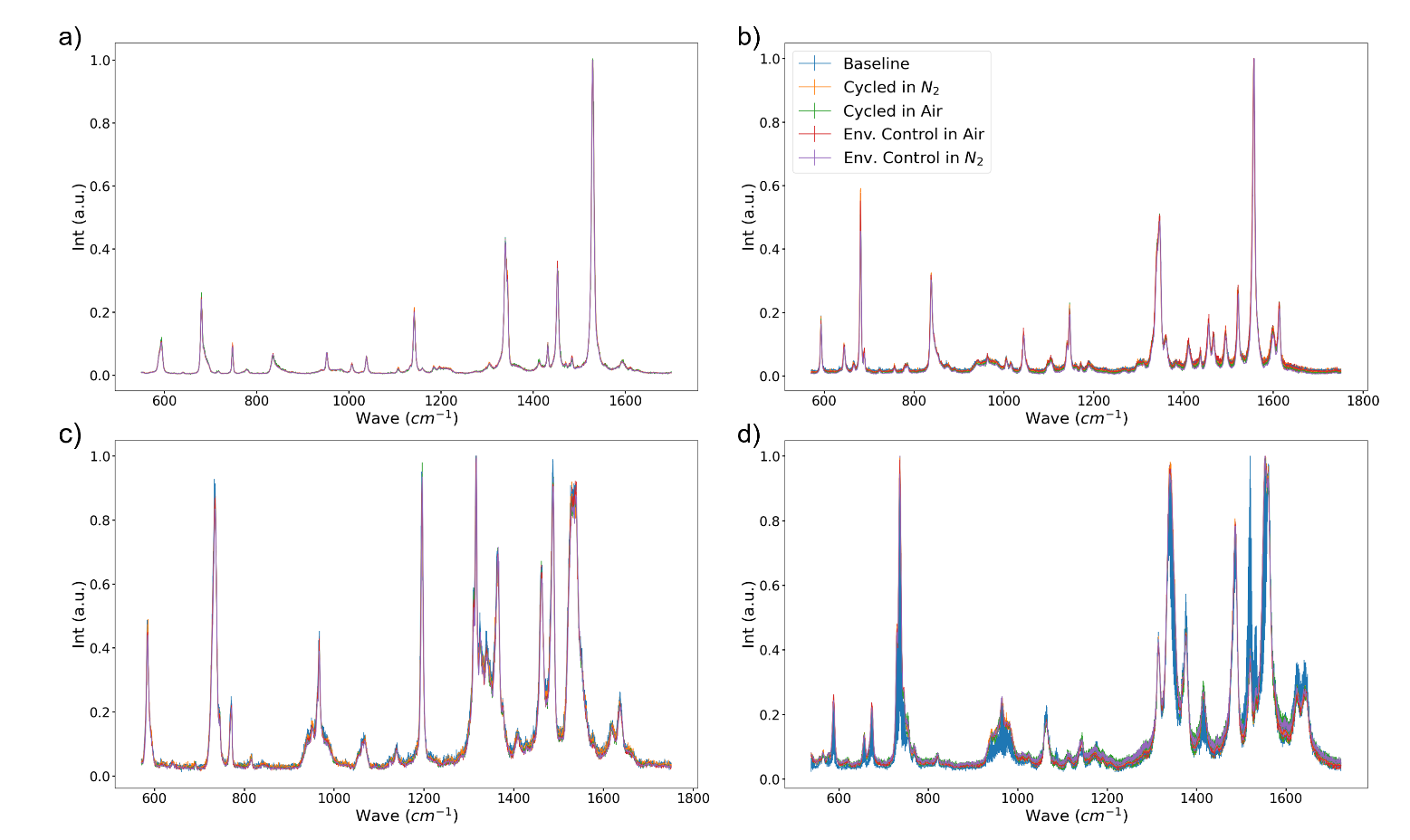
*

**Figure S11**) Raman Spectra of the CuPc (a), F_10_SiPc (b), F_16_CuPc (c) and (F_5_PhO)_2_-F_16_-SiPc (d), baseline, cycling and Env. Control devices.

*
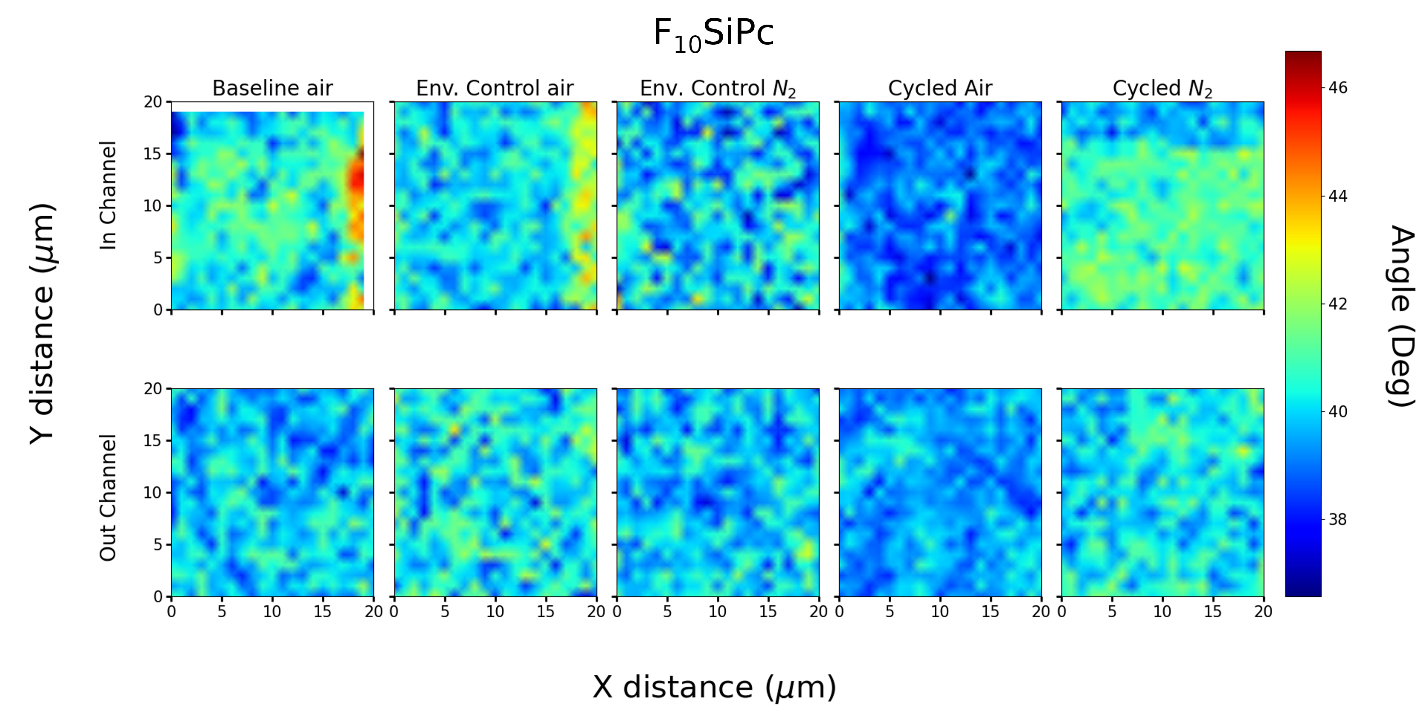
*

**Figure S12**) 2D molecular orientation maps in the channel and out of the channel of F_10_SiPc Baseline, Env. Control and cycled devices. Maps were made using polarized Raman microscopy.

*
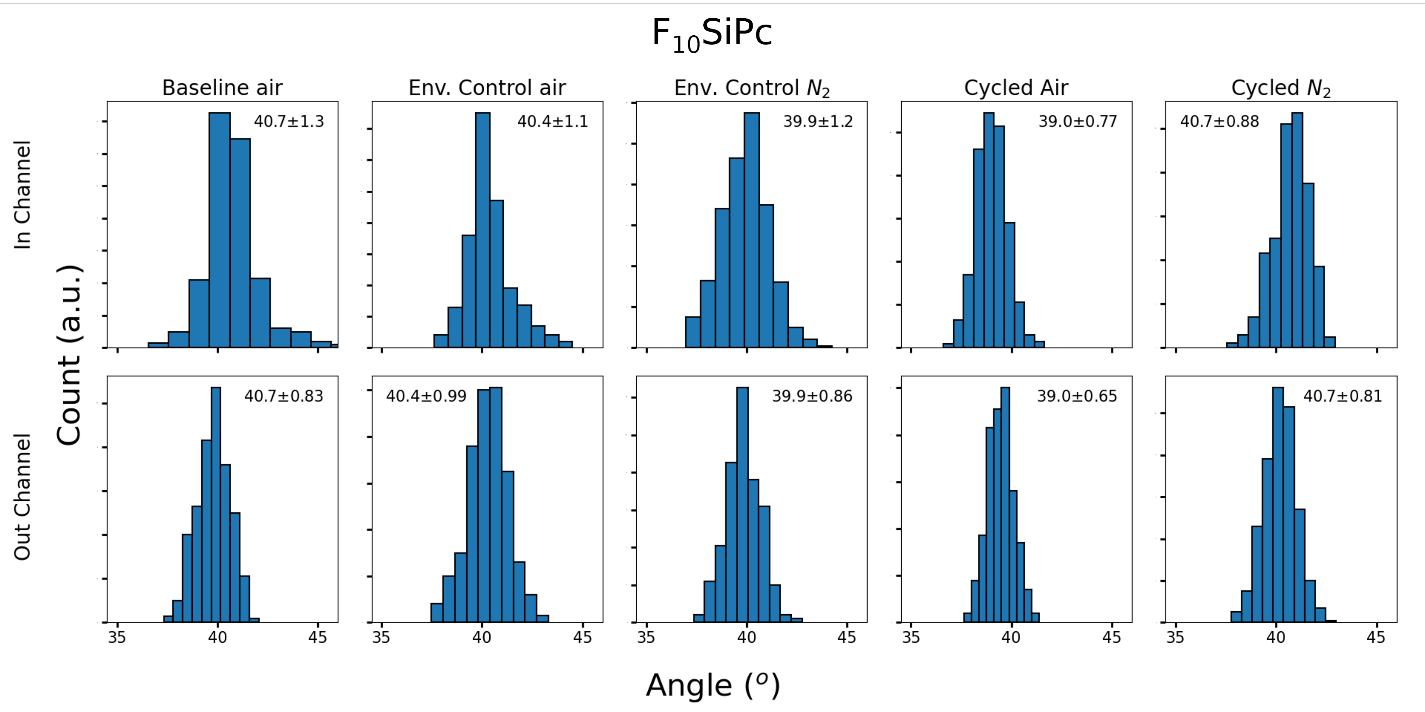
*

**Figure S13**) Histograms of the 2D molecular orientation maps in the channel and out of the channel of F_10_SiPc Baseline, Env. Control and cycled devices from Figure S6.

*
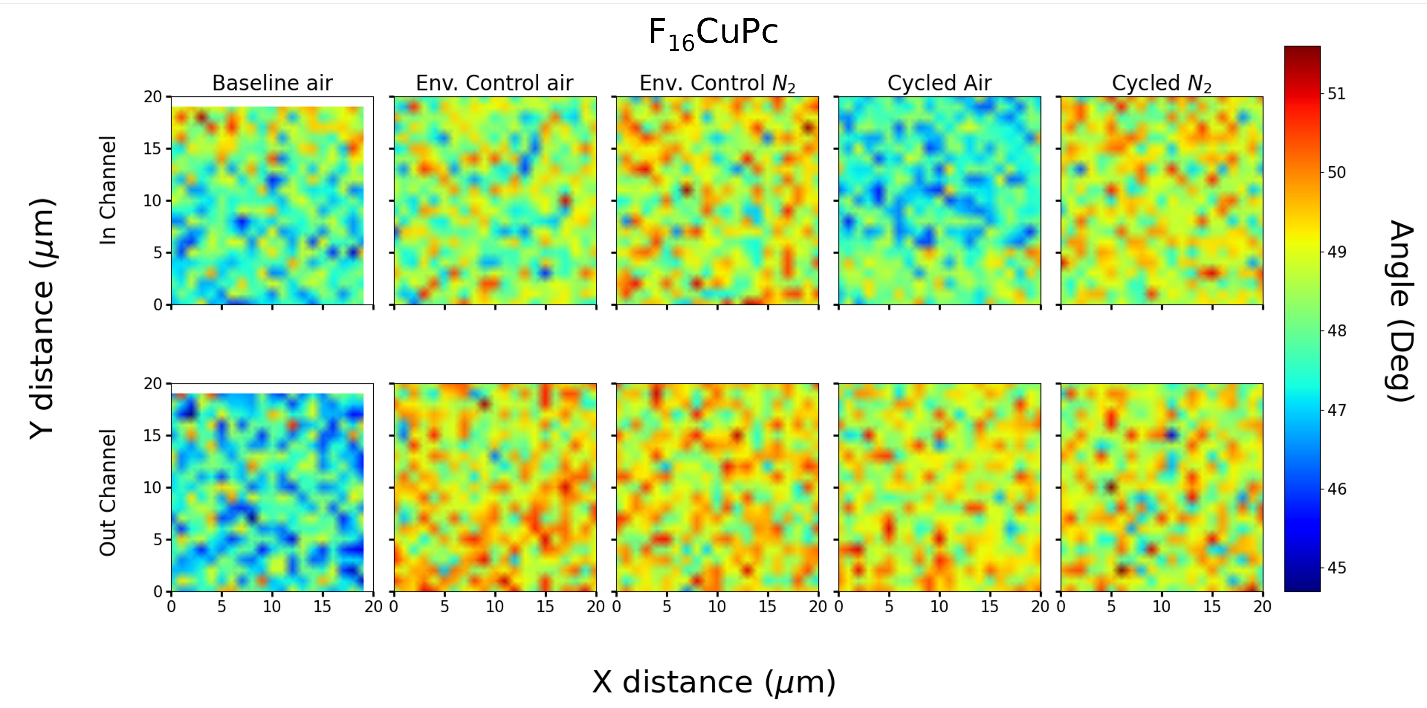
*

**Figure S14**) 2D molecular orientation maps in the channel and out of the channel of F_16_CuPc Baseline, Env. Control and cycled devices. Maps were made using polarized Raman microscopy.

*
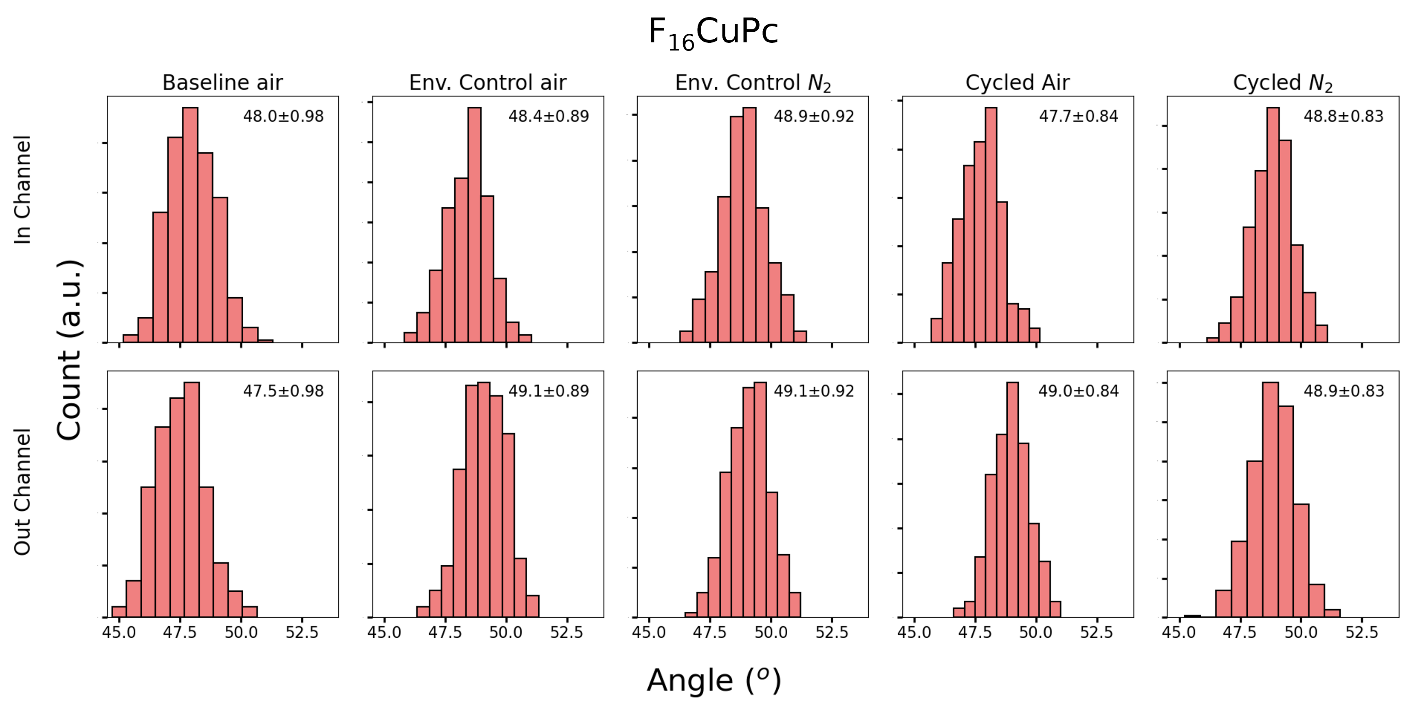
*

**Figure S15**) Histograms of the 2D molecular orientation maps in the channel and out of the channel of F_16_CuPc Baseline, Env. Control and cycled devices from Figure S8.

*
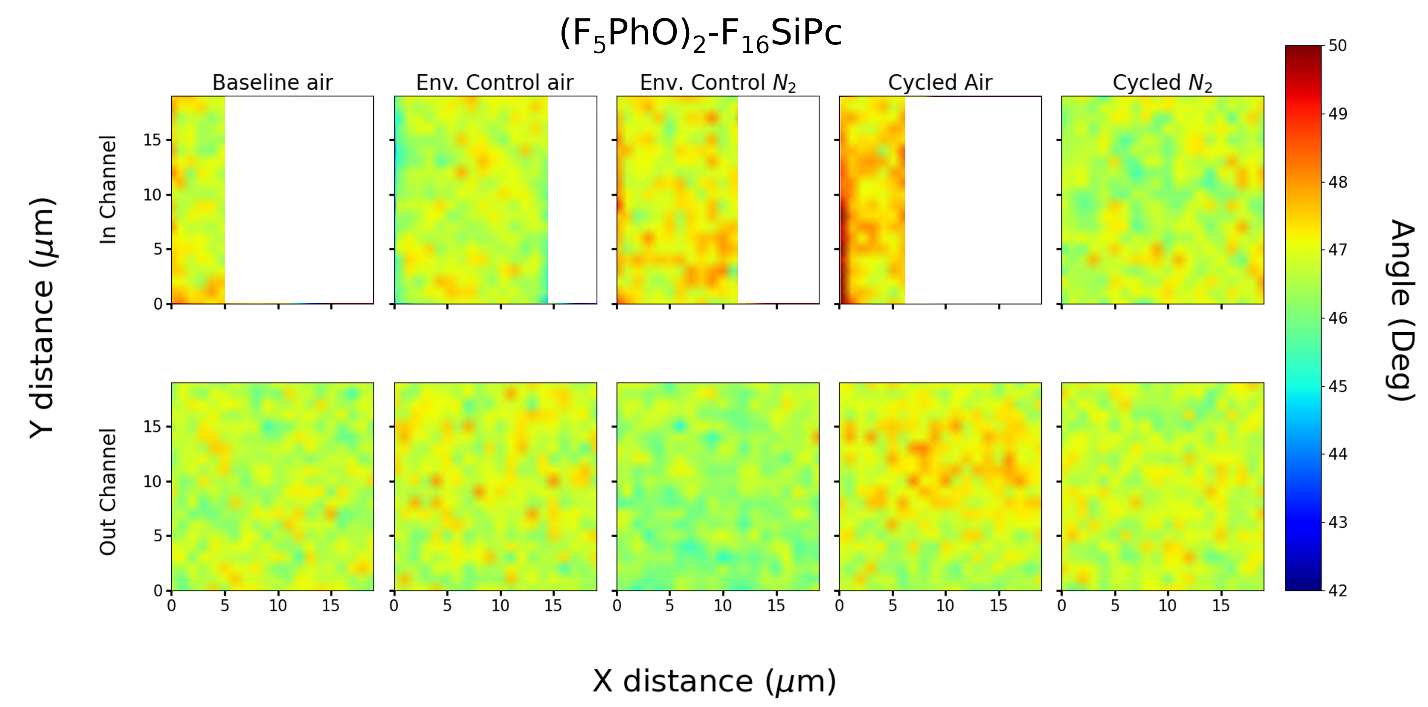
*

**Figure S16**) 2D molecular orientation maps in the channel and out of the channel of (F_5_PhO)_2_-F_16_-SiPc Baseline, Env. Control and cycled devices. Maps were made using polarized Raman microscopy.

For the (F_5_PhO)_2_-F_16_-SiPc, some of the channel electrodes overshadowed the semiconductor, causing discrepancies from either side. This shadowing was not visible via optical microscope and was only visible after developing the Raman molecular orientation maps. Therefore, the maps in the channel were fixed to remove these discrepancies.

*
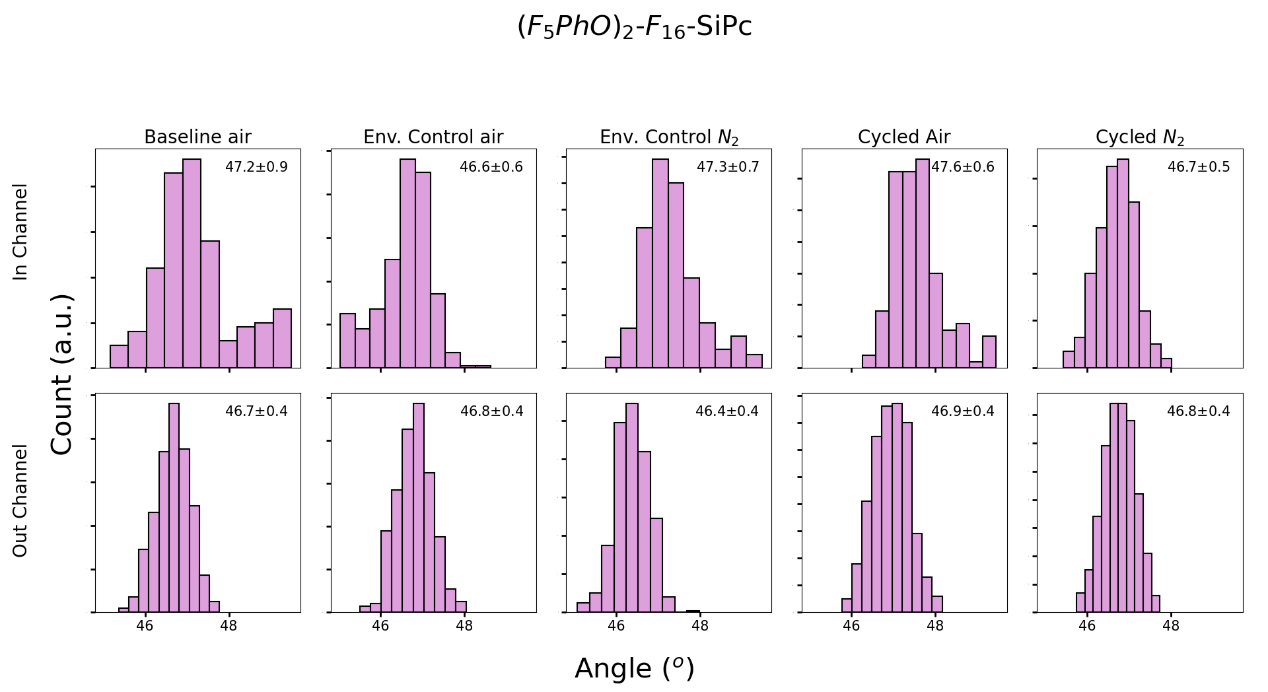
*

**Figure S17**) Histograms of the 2D molecular orientation maps in the channel and out of the channel for (F_5_PhO)_2_-F_16_-SiPc Baseline, Env. Control and cycled devices from Figure S10.

**Table S2**) Model output parameters extracted from model fittings in Figure S8

|  | | **N_2_** | | | **Air** | | |
| --- | --- | --- | --- | --- | --- | --- | --- |
|  |  | **Baseline** | **Env. Control** | **Cycled** | **Baseline** | **Env. Control** | **Cycled** |
| **CuPc**  ^a)^ | *μ_h_* ^b)^ **_p_** | 0.0115 | 0.0120 | 0.0129 | 0.0178 | 0.0178 | 0.02 |
|  | *H_D_* ^b)^ | 3x10^18^ | 3 x10^18^ | 5 x10^18^ | 3 x10^18^ | 3 x10^18^ | 3 x10^18^ |
|  | *T_CD_* ^b)^ | 1550 | 1550 | 1550 | 2000 | 2000 | 2000 |
|  | *N*_int_ ^b)^ | 5.5x10^11^ | 8.8 x10^11^ | 9 x10^11^ | -9 x10^11^ | -3.5 x10^12^ | -3.5 x10^12^ |
| **F_10_SiPc**  ^a)^ | *μ_e,_ μ_h_* ^b)^ | 0.200 | 0.190 | 0.099 | 0.0264 | 0.0696 | 0.0466 |
|  | *H_A,_ H_D_* ^b)^ | 1 x10^18^ | 1 x10^18^ | 1 x10^18^ | 5 x10^18^ | 5 x10^18^ | 5 x10^18^ |
|  | *T_CA,_ T_CD_* ^b)^ | 1400 | 1400 | 1400 | 1800 | 1800 | 1800 |
|  | *N*_int_ ^b)^ | -3.1 x10^11^ | -7.2 x10^11^ | -7 x10^11^ | 2 x10^12^ | 1.5 x10^12^ | 1.5 x10^12^ |
| **F_16_CuPc**  ^a)^ | *μ_e,_* ^b)^ | 0.0855 | 0.0855 | 0.0855 | 0.0164 | 0.00411 | 0.0224 |
|  | *H_A_* ^b)^ | 6 x10^18^ | 6 x10^18^ | 6 x10^18^ | 5 x10^18^ | 5 x10^18^ | 3 x10^18^ |
|  | *T_CA_* ^b)^ | 1400 | 1400 | 1400 | 1400 | 1400 | 1400 |
|  | *N*_int_ ^b)^ | -9 x10^10^ | -9 x10^10^ | -9 x10^10^ | -1 x10^11^ | -5 x10^11^ | -2.7 x10^12^ |
| **(F_5_PhO)_2_-F_16_-SiPc**  ^a)^ | *μ_e_* ^b)^ | 0.0340 | 0.0450 | 0.0540 | 0.0114 | 0.00745 | 0.0103 |
|  | *H_A_* ^b)^ | 5 x10^18^ | 5 x10^18^ | 5 x10^18^ | 5 x10^18^ | 5 x10^18^ | 5 x10^18^ |
|  | *T_CA_* ^b)^ | 2000 | 2000 | 2200 | 2000 | 2200 | 2000 |
|  | *N*_int_ ^b)^ | 1 x10^12^ | 5 x10^11^ | 5 x10^11^ | -1 x10^11^ | -2 x10^12^ | -5 x10^11^ |

a) Bottom gate top contact OTFTs made with Copper phthalocyanine (CuPc), Copper(II) 1,2,3,4,8,9,10,11,15,16,17,18,22,23,24,25-hexadecafluoro-29H,31H-phthalocyanine (F_16_CuPc), silicon bis(pentafluorophenoxy)phthalocyanine (F_10_SiPc) and ((F_5_PhO)_2_-F_16_-SiPc) as the semiconductor, characterized as either p-type or n-type either in Air or N2 (Env.). The Test type refers to if the corresponding OTFT was characterized baseline (or pristine device), the same device after being continuously cycled as an OTFT for 42-72 h (CuPc = 71.1 h, F16CuPc = 41.9 h, F10SiPc = 47.8 h and (F5PhO)2-F16-SiPc = 48.6 h) labeled as cycled, or the control devices under the same environment characterized after 42-72 h labeled Env. Control. b) N_int_ : interface charge density [cm^–2^], μ_h_, μ_e_: hole and electron mobility [cm^2^/ Vs], H_D_, H_A_ : donor/acceptor-like exponential trap density [cm^-3^], T_CD_, T_CA_ : donor/acceptor-like exponential trap temperature [K].
